# Treatment of women with postpartum mental disorders in a day clinic mother-baby unit and the effect on child behavioural problems – A 1-year follow-up

**DOI:** 10.1016/j.ijchp.2025.100587

**Published:** 2025-06-11

**Authors:** Susann Steudte-Schmiedgen, Luisa Bergunde, Julia Frohberg, Antje Bittner, Anne Coenen, Susan Garthus-Niegel, Juliane Junge-Hoffmeister, Kerstin Weidner

**Affiliations:** a Department of Psychotherapy and Psychosomatic Medicine, Faculty of Medicine and University Hospital Carl Gustav Carus, TUD Dresden University of Technology, Dresden, Germany; b Institute and Policlinic of Occupational and Social Medicine, Faculty of Medicine and University Hospital Carl Gustav Carus, TUD Dresden University of Technology, Dresden, Germany; c Institute for Systems Medicine (ISM), Faculty of Medicine, Medical School Hamburg, Hamburg, Germany; d Department of Childhood and Families, Norwegian Institute of Public Health, Oslo, Norway

**Keywords:** Postpartum mental disorders, Mother-baby unit, Day clinic, Long-term effects, Psychotherapy, Offspring behaviour problems

## Abstract

**Background:**

Postpartum mental disorders are highly prevalent with substantial impact on mother-child bonding and child development. While short-term benefits of an interaction-focused mother-baby treatment for maternal mental health are documented, little is known about the stability of these effects and their influence on child behavioural development.

**Method:**

This prospective study included 348 women with postpartum mental disorders who received dyadic treatment at a specialized mother-baby day clinic. Maternal symptoms of depression (EPDS), anxiety (STAI-T), overall psychological distress (BSI-GSI) as well as parenting sense of competence (PSOC) were assessed at admission, discharge, and 1-year follow-up, along with diagnostic classification at admission. At 1-year follow-up, mothers (*n* = 164) completed the Child Behaviour Checklist (CBCL) to measure child behavioural problems.

**Results:**

Maternal psychopathology and PSOC improved significantly from admission to discharge, with clinically meaningful effects. No additional improvements emerged from discharge to 1-year follow-up, except for a tentative reduction in anxiety symptoms. All outcome measures and outcome trajectories regarding anxiety symptoms and overall psychological distress varied by primary clinical diagnosis. Greater maternal symptom improvement from admission to 1-year follow-up was associated with fewer child behavioural problems. However, this effect was not found for symptom changes from admission to discharge when controlling for maternal symptoms at 1-year follow-up.

**Conclusions:**

Interaction-focused treatment in a mother-baby day clinic may be associated with clinically meaningful improvements in maternal mental health outcomes up to 1-year follow-up. These long-term improvements may also relate to less child behavioural problems. However, the absence of a waiting list control group warrants cautious interpretation of findings.

## Introduction

Postpartum mental disorders are highly prevalent, affecting up to one in five mothers (Howard & Khalifeh, 2020). Specifically, postpartum depression has been reported in 14–18 % of women (Hahn-Holbrook et al., 2018; Liu et al., 2022; Shorey et al., 2018), anxiety disorders in 9–15 % (Dennis et al., 2017; Goodman et al., 2016) and substance use disorders in 12–15 % (Vesga-Lopez et al., 2008). Other less frequent disorders include post-traumatic stress disorder (3–5 %, Heyne et al., 2022; Yildiz et al., 2017), borderline personality disorder (1–2 %, Prasad et al., 2022), and psychosis (0,5 %, Vesga-Lopez et al., 2008). Postpartum mental disorders significantly impact the mother-child relationship and child development (Howard & Khalifeh, 2020). For example, a longitudinal study in a large UK sample showed that persistent and severe postpartum depression increases the risk for adverse psychological outcomes in offspring, including behavioural problems at 3.5 years of age and a higher risk of depression at 18 years of age (Netsi et al., 2018). The negative effects of postpartum mental disorders on the psychological, but also physical development of the child have been replicated in numerous studies (reviewed in Pierce et al., 2020; Stein et al., 2014). Despite their high prevalence, at least half of the cases of postpartum mental disorders are not detected, and only a small proportion of identified women receive evidence-based, specialized treatment (Bauer et al., 2014; Marcus, 2009).

In the early postpartum period, a *dyadic* mother-child therapy is a promising treatment option for various maternal postpartum mental disorders. A basic assumption for a dyadic treatment is that the early mother-child relationship is critical to the child's future health and well-being. Mental disorders can negatively influence parental behaviour with corresponding negative effects on the mother-child relationship and the child's attachment development (Erickson et al., 2019). Thus, in addition to ameliorating maternal psychopathological symptoms, the dyadic treatment aims to promote mother-child bonding, maternal sensitivity, learning to meet the baby's needs, and the experience of parental competence (Bittner et al., 2021; Weidner et al., 2021). Previous studies have provided evidence that early dyadic mother-baby treatment is effective (Howard & Khalifeh, 2020). Specifically, such interventions have been shown to increase parental sensitivity and improve child attachment security (e.g., Bakermans-Kranenburg et al., 2003; Mascheroni & Ionio, 2019; Mountain et al., 2017; Tsivos et al., 2015). The positive effects of early dyadic treatment on maternal symptomatology and the mother-child relationship have been documented in studies of inpatients in mother-baby units (Christl et al., 2015; reviewed in Connellan et al., 2017; Glangeaud-Freudenthal et al., 2011; Salmon et al., 2003). This is also in line with data from a day clinic mother-baby unit (MBU) observing improvements in maternal psychological symptoms, maternal bonding, and parenting outcomes from admission to discharge with large effect sizes (Weidner et al., 2021). Interestingly, improvement levels regarding symptoms of depression and overall psychological distress varied by clinical diagnosis, with women with personality disorders showing elevated scores at admission and less symptom reduction compared to other diagnostic groups (i.e., depressive disorders, anxiety disorders, schizophrenia/bipolar disorders). Women with depressive disorders scored the highest in bonding difficulties as well as lowest in parental self-efficacy at admission and showed the greatest improvements in bonding difficulties as compared to the other diagnostic groups (i.e., anxiety disorders, personality disorders, schizophrenia/bipolar disorders).

An open question is whether these treatment effects remain stable over longer time periods. A review and meta-analysis including randomized controlled trials (RCT) revealed that the benefits of psychological treatment of postpartum depression were not maintained at later follow-ups (Huang et al., 2020). However, previous research in this context primarily focused on the outpatient setting and postpartum depression (e.g., Cooper et al., 2003; reviewed in Huang et al., 2020) or examining the effect of a single intervention (e.g., Kersten-Alvarez et al., 2010). The few follow-up studies on the impact of MBU care are limited to an inpatient setting (reviewed in Connellan et al., 2017; e.g., Vliegen et al., 2010). Hence, there is a lack of data on the long-term benefits of a lower-threshold day clinic treatment. Furthermore, more research is needed to examine whether MBU treatment is associated with a preventive effect on offspring’s behavioural development (e.g., Aktar et al., 2019; Poobalan et al., 2007). Previous RCT studies in an outpatient setting suggest that treating either postpartum maternal depression or the parent-infant relationship alone is insufficient to positively impact child development (Aktar et al., 2019; Forman et al., 2007; Murray et al., 2003; Nylen et al., 2006; Poobalan et al., 2007). Therefore, targeting both maternal psychopathology *and* the mother-baby relationship is likely crucial to mitigate the adverse effects of postpartum mental disorders on child development (Aktar et al., 2019; but see Stein et al., 2018). Together, while previous studies have predominantly focused on outpatient or inpatient settings, single interventions, or short-term follow-ups in rather homogeneous clinical samples mostly suffering from postpartum depression, this study aims to offer new insights by investigating symptom trajectories in the context of a lower-threshold day clinic dyadic treatment in a mixed clinical sample (Weidner et al., 2023) and potential preventive effects on offspring's behavioural development.

This prospective study included 348 women with postpartum mental disorders who received specialized treatment focusing on mother-child interaction offered by a day clinic, including 1-year follow-up data. The study aimed to examine the long-term course of maternal psychological symptoms (i.e., depression, anxiety, general psychological distress) and parenting sense of competence from admission to discharge up to 1-year follow-up. It was hypothesized that improvements in clinical outcomes observed from admission to discharge (Weidner et al., 2021) remained stable at 1-year follow-up. Further, the study set to extend prior findings by investigating whether symptom severity and trajectories up to 1-year follow-up were affected by mothers` primary clinical diagnosis (Weidner et al., 2021). Finally, it was expected that improvements in maternal clinical symptoms were associated with less child behavioural problems at the 1-year follow-up.

## Material and methods

### Participants and procedure

Data were used from mothers who received an interaction-focused MBU treatment at the day clinic of the Department of Psychotherapy and Psychosomatic Medicine at the University Hospital Carl Gustav Carus of the TUD Dresden University of Technology between 2010 and 2021. MBU admission criteria include severe postpartum mental disorders, as determined by clinical judgement and standardized questionnaires, requiring an intensive day clinic intervention. Exclusion criteria for MBU treatment comprise psychopathological symptoms making an inpatient psychiatric treatment necessary (e.g., acute psychosis, severe affective disorder with manic symptoms or suicidality) as well as characteristics of the baby (e.g., prematurity, other reasons for high susceptibility to infection) preventing participation in a mother-baby treatment in a group setting. Further exclusion criteria of the study included inadequate German language skills and age < 18 years. In addition, patients who received MBU treatment for a second time were excluded. At admission, diagnostic classification of mothers was ensured by trained and supervised psychotherapists of the MBU during the admission consultation according to ICD-10 diagnostic criteria (World Health Organization, 2004) and verified using a structured clinical interview for DSM-IV disorders (SCID-I/SCID-II, Wittchen et al., 1997).

The interaction-focused mother-child treatment provided by the MBU offers all modules of a complex interdisciplinary psychosomatic treatment (Bittner et al., 2021; Weidner et al., 2021). A particular focus is on video-based interaction therapy with recordings of standardized mother-child interaction situations (e.g., baby massage, play and feeding situations, changing diapers) during the course of MBU treatment. The aim is to make positive but also critical interactions visible, to promote intuitive parental competence, to train a resource-oriented view, and to make the positive progress visible. Other treatment components include among others group therapy, body therapy, and disorder-specific individual therapy. Treatment duration encompasses on average four days per week over the course of eight weeks.

All women treated at the MBU underwent a routine assessment procedure, completing standardized questionnaires (at admission, discharge, 1-year follow-up) and self-developed questions regarding sociodemographic- and birth-related aspects. All participants provided written informed consent.

A sample of *N* = 348 mothers took part in at least one measurement occasion (intent-to-treat sample), with *n* = 341 women providing data at treatment admission, *n* = 307 women providing data at discharge, and *n* = 167 women providing additional data at the 1-year follow-up assessment. Of these women, *n* = 323 (92.8 %) completed day hospital treatment and *n* = 25 (7.2 %) had an irregular termination of treatment due to varying reasons (e.g., patient’s wish, separation of mother and child due to children being taken into custody, COVID-19 pandemic). Of the latter group, 96.0 % of women still participated at admission while 32.0 % participated at discharge and 32.0 % at follow-up.

Dropout analyses for the intent-to-treat sample were conducted with Welch-Test and Fischer’s exact test to examine differences between *n* = 167 participants who participated at the 1-year follow-up assessment (48 %) and *n* = 181 who did not (52 %). Results indicated no differences regarding sociodemographic aspects, psychopathological symptom severity, parenting competence, primary clinical diagnosis and maternal bonding, or change in symptoms from admission to discharge (see supplementary materials A). However, women who did not participate at 1-year follow-up showed significantly higher levels of depression symptoms at discharge ((*M* = 10.67 (*SD* = 5.15) vs. *M* = 8.90 (*SD* = 4.48); *t* (293.31) = −3.19, *p* = .002) and had a shorter treatment duration (*M* = 8.23 weeks (*SD* = 2.93) vs. *M* = 8.95 weeks (*SD* = 2.53); *t* (344.59) = 2.44, *p* = .015). Among women who did not participate at 1-year follow-up, *n* = 17 (9.4 %) had an irregular treatment termination.

### Instruments

#### Edinburgh postnatal depression scale (EPDS)

Depressive symptoms were assessed using the Edinburgh Postnatal Depression Scale (EPDS - German version: Bergant et al., 1998; Cox et al., 1987). The EPDS is a reliable self-report questionnaire asking about the severity of ten symptoms over the past week with four response categories ranging from 0 to 3 (total score: 0 to 30). Higher total scores indicate higher symptom severity. A cut-off of ≥ 10 for probable minor depression was used (Weigl & Garthus-Niegel, 2021).

#### State-Trait anxiety inventory – trait subscale (STAI-T)

The trait subscale of the State-Trait Anxiety Inventory (STAI-T) was used to measure interindividual differences in an anxious disposition (German version: Laux, 1981; Spielberger et al., 1970). The scale consists of 20 items being rated on a 4-point-Likert scale. A cut-off of ≥ 47 for clinically relevant anxiety was applied. The STAI shows excellent reliability criteria and standard values and has been validated for different conditions (Laux, 1981).

#### Brief symptom inventory (BSI-GSI)

The BSI was used to measure general psychopathology during the last seven days with 53 items on a 5-point Likert scale (ranging from 0 “not at all” to 4 “very much”; Derogatis, 1993; Franke, 2000). The global severity index (GSI) of the BSI reflects the overall level of distress due to mental and somatic issues. The GSI is calculated as the mean of the single item scores and may range between 0 and 4. The cut-off of ≥ 0.63 indicating a clinically relevant amount of distress for this scale was used. Reliability of the BSI-GSI is excellent and convergent and discriminant validity have been shown (Franke, 2000).

#### Parenting sense of competence scale (PSOC)

The PSOC assesses parenting competence on two dimensions: satisfaction and efficacy (Johnston & Mash, 1989). It is a 16 item Likert-scale questionnaire (6-point scale ranging from “1 - strongly agree” to “6 - strongly disagree”). The satisfaction subscale (9 items) measures parents’ anxiety, motivation, and frustration, while the efficacy subscale (7 items) assesses parents’ competence, capability levels, and problem-solving abilities in the parental role (Johnston & Mash, 1989). Reliability of the PSOC is satisfactory and factorial validity has been shown (Johnston & Mash, 1989; Ohan et al., 2000).

#### Parental bonding questionnaire (PBQ)

The PBQ was used to assess the maternal bonding of the mother toward her baby (Brockington et al., 2001) at admission and discharge. The PBQ consists of 25 items in which mothers are asked to think of the most challenging time with their baby and to rate specific experiences on a scale ranging from 0 (“never”) to 5 (“always”). While originally four factors were proposed (“impaired bonding”, “anxiety about care”, “lack of enjoyment and affection for the baby”, and “rejection and risk of abuse”; Brockington et al., 2001), this could not be replicated in German samples, thus supporting the use of one general factor calculated as the sum of item scores (e.g., Göbel et al., 2024; Reck et al., 2006; Weigl & Garthus-Niegel, 2021). Higher values thus indicate a higher extent of mother-child bonding impairment (range 0 – 125). A cut-off for the total score with ≥ 26 for a probable bonding disorder and ≥ 40 for a severe bonding disorder were used (Brockington et al., 2006).

#### Child Behaviour Checklist (CBCL)

The CBCL for ages 1.5 – 5 years, which was completed by mothers at 1-year follow-up, was used to assess behavioural problems of their children, e.g., attention problems, aggressive behaviour, somatic complaints, anxiousness/depressiveness (Achenbach, 1991). The CBCL consists of 100 items with three possible responses (from 0 “not applicable” to 1 “somewhat or sometimes applicable” to 2 “accurate or often applicable”). For the purpose of this study, we calculated a sum score from all items as well as the internalizing and externalizing subscales. The CBCL shows good reliability (Achenbach & Rescorla, 2000).

#### Sociodemographic and birth-related information

At admission, mothers self-reported their age (in years), their child’s age (in months), the gestational week, whether they had more than one child (one child vs. more than one child), their level of school education (achieved German high school diploma or not), whether they were a single parent (yes/no), how supported they feel regarding the care of their child (supported = “very well supported” or “rather well supported”; not supported = “rather not supported” or “not at all supported”), and how satisfied they feel with their financial situation (satisfied = “completely satisfied” or “rather satisfied”; not satisfied = “rather not satisfied” or “not at all satisfied”) and birth mode (vaginal birth including spontaneous, with induction, and operative; caesarean section, including planned and emergency).

### Statistical analyses

Data were analysed using the statistical package SPSS, version 29 (SPSS Inc., Chicago, IL, USA) and R version 4.4.2 (R Core Team, 2023). Analyses were tested two-sided, and a 5 % significance level was applied. For questionnaire data, missing values were replaced with the mean of the participant’s completed items if no >10 % of responses were missing (Graham, 2009; Newman, 2014). Specifically, a mean score was calculated by summing the available responses to the individual items on the respective scale and dividing by the number of completed items. This approach was applied to between 0.6 % and 14 % of participants across the three assessment points (EPDS: 0.6 %–1.1 %, STAI-T: 1.2 %–4.1 %, BSI-GSI: 7.5 %–14 %, PSOC: 2.3 %–6.1 %). All subsequently described analyses were conducted for each of the psychopathological and parenting outcome variables (i.e., EPDS, STAI-T, BSI-GSI, PSOC).

Intent-to-treat analyses were performed including *N* = 348 mothers. Linear mixed effect regressions with random intercept parameters were calculated with the *lme4* package with maximum likelihood estimation (ML; Hoffman, 2015; Sharpe & Cribbie, 2023). First, we estimated an empty means, random intercept model to calculate the intraclass correlation coefficient (ICC), which gives the proportion of variance in the respective outcome that is due to constant mean differences between individuals. Second, to examine the change in clinical outcomes across treatment, we added a fixed effect of Time as a categorical within-subjects factor with three levels (admission (0), discharge (1), and 1-year follow-up (2)) as well as treatment duration as a between-subjects continuous control variable. Third, for analyses examining the effect of Group and Group*Time interaction, we excluded individuals (*n* = 29) whose primary diagnosis could not be grouped into one of the four categories of 1) depressive disorders, 2) anxiety disorders including obsessive compulsive disorders (OCD) and posttraumatic stress disorders (PTSD), 3) personality disorders, and 4) schizophrenia/bipolar disorder, resulting in *N* = 319 individuals’ data being used. First, in addition to Time and treatment duration, the fixed effect of Group was added as a between-subjects factor with four categories. In a next step, we added the interaction between Group and Time as a fixed effect. We used the *emmeans* package to examine post-hoc comparisons for Time, Group, and specific interaction contrasts, to which we applied a false discovery rate (FDR) correction for multiple testing throughout. Plots were created with the *ggplot2* package. Due to 52 % dropout from admission to 1-year follow-up, we checked missingness patterns and found treatment duration to significantly predict missingness. To accommodate the Missing at Random (MAR) assumption, we included this variable in subsequent analyses. Also, we conducted sensitivity analyses with multiple imputed data. We used multiple imputation by chained equations (MICE) as implemented in the *mice* package in R (van Buuren & Groothuis-Oudshoorn, 2011) as recommended when data contain more 10 % partial responders (Newman, 2014; details in supplementary materials D). The overall pattern of results remained the same. Any changes in the effects are noted in the results section and these findings are discussed carefully.

We also computed the reliable change index (RCI; Jacobson & Truax, 1991) for all outcome measures to infer whether the changes observed across time are reliable (i.e., not only statistically, but also clinically significant; Guhn et al., 2014). To do so, the RCI is calculated as a ratio where the numerator represents the actual observed difference between two measurements and the denominator is the standard error of measurement of the difference, taking account of the measure’s reliability and the standard deviation at admission. This formula is then solved for RCI at 1.96, with the result representing the minimum difference between measurement points that is necessary to be 95 % confident that the observed change is reliable and not solely attributable to measurement error (Guhn et al., 2014).

To investigate the effect of change in clinical outcomes across treatment on child behavioural problems (i.e., CBCL sum score, CBCL internalizing symptoms, CBCL externalizing symptoms), we conducted (hierarchical) multiple linear regression analyses in R using the *stats* package. Regarding the effect of change in clinical outcome from admission to discharge, we added the level of the clinical measure at admission, treatment duration, and the change score for each clinical outcome from admission to discharge as independent variables in model 1 and then added maternal symptoms in the respective outcome at 1-year follow-up in model 2. Regarding the effect of change in clinical outcome from admission to 1-year follow-up, the level of the clinical measure at admission and treatment duration were added in addition to the change score of the clinical measures.

## Results

### Descriptive findings

Baseline sociodemographic, birth-related and clinical characteristics of the sample are shown in Table 1. The sample included *N* = 348 mothers who were on average 29.83 years old at admission. The children were on average 24.05 weeks old at admission and 66.9 % of mothers had only one child. About a third of women were married and another third had sole custody of their child. Additionally, 70.6 % reported somatic illnesses (e.g., psoriasis, diabetes mellitus, hypothyroidism, irritable bowel syndrome, asthma) and 80.4 % reported taking medication during day clinic stay. Of the latter, 71.4 % reported the intake of psychotropic medication. Primary clinical diagnoses comprised depressive disorders (38.8 %), anxiety disorders including OCD and PTSD (27.9 %), personality disorders (20.1 %) as well as schizophrenia and bipolar disorders (4.9 %). Twenty-nine women (8.3 %) had heterogeneous clinical diagnoses such as eating disorders, somatoform disorders, substance use disorders and were categorized in an “other” group. On average, women had 1.86 comorbid F-diagnoses according to ICD-10. Additional descriptive information on mothers according to their diagnostic status is provided in the supplementary materials B.

**Table 1:** Sociodemographic, clinical and birth-related characteristics of mothers (*N* = 348).

	Mean (*SD*, range) or n (%)
*Sociodemographic information*	
Age mother in years	29.83 (5.68, 18 – 45)
Age child in weeks	24.05 (13.48, 2 – 82)
Gestational week	39.55 (2.26, 23 – 46)
Preterm birth	24 (10.4)
Birth mode	
**-** Vaginal birth	208 (79.4)
**-** Caesarean section	54 (20.6)
Average number of children	1.49 (0.87, 1 – 6)
Number of children **-** One child **-** Two children **-** Three children **-** Four and more children	196 (66.9) 68 (23.2) 18 (6.1) 11 (3.7)
Marital status **-** Not married **-** Married **-** Divorced **-** Widowed	216 (65.5) 96 (29.1) 17 (5.2) 1 (0.3)
Single mother	79 (24.6)
Child custody **-** Mother **-** Mother and father	97 (30.1) 225 (69.9)
Education **-** High school diploma **-** Primary and lower secondary education	138 (41.8) 192 (58.2)
*Clinical information*	
Length of MBU stay in weeks	8.58 (2.76, 0.5 – 18.5)
Primary clinical diagnoses **-** Depressive disorders **-** Anxiety disorders (including OCD and PTSD) **-** Personality disorders **-** Schizophrenia or bipolar disorder **-** Others (e.g., eating disorders, somatoform disorders, substance use disorders)	135 (38.8) 97 (27.9) 70 (20.1) 17 (4.9) 29 (8.3)
Number of mental comorbidities	1.86 (1.29, 0 – 6)
Somatic diseases (self-report)	240 (70.6)
Medication intake during day clinic stay **-** Psychotropic medication **-** Other medication	271 (80.4) 192 (71.4) 177 (65.8)
Parent-child bonding (PBQ)	
**-** At admission	30.96 (21.53, 0 – 98)
Above cut-off	177 (53.0)
**-** At discharge	15.14 (10.07, 0 – 53)
Above cut-off	39 (12.8)
*1-year follow-up information*	
Child behaviour problems (CBCL) at 1-year follow-up	
**-** Total score	25.37 (14.85, 1 – 81)
Above cut-off	6 (3.7)
**-** Externalizing symptoms	10.85 (6.52, 0 – 34)
Above cut-off	14 (8.5)
**-** Internalizing symptoms	5.46 (4.07, 0 – 21)
Above cut-off	7 (4.3)
Continued treatment or received assistance after discharge (self-report at 1-year follow-up)^a^	152 (91.6)

Notes. *N* varied slightly due to missing values. OCD = Obsessive compulsive disorder, PTSD = Posttraumatic Stress Disorder, PBQ = Parental Bonding Questionnaire, CBCL = Child Behaviour Checklist, MBU = mother baby unit.

a outpatient psychotherapy at the University Hospital / with a registered therapist, medication treatment at the University Hospital / with a registered doctor, inpatient treatment, day clinic treatment, outpatient mother-child bonding work (e.g., occupational therapy), extended midwife assistance, domestic help, or support from the youth welfare office.

### Clinical outcomes over the course of day clinic MBU treatment up to 1-year follow-up

Table 2 shows maternal treatment and parenting outcomes for all measurement points as well as the ICC values from the empty means, random intercept model suggesting that between 17 % and 38 % of the variance in clinical and parenting outcomes is due to stable between-person differences (trait), whereas 62 % to 83 % is due to within-person (state) factors. Analyses showed a significant effect for time for all clinical and parenting measures with FDR-corrected pairwise comparisons revealing a significant decrease from admission to discharge (all *p’s* < 0.001: EPDS *b* = 6.15; STAI-T *b* = 8.55; BSI-GSI *b* = 0.56; PSOC *b* = −8.59). No significant additional changes from discharge to 1-year follow-up were observed (*p*’s > .307), apart from a significant decrease in STAI-T (STAI-T *b* = 1.60, *p* = .035). However, the latter finding was not confirmed by sensitivity analyses using multiple imputed data (*b* = 0.93, *p* = .245; see supplementary materials D). Treatment duration had a significant main effect on average STAI-T (*b* = 0.39, *p* = .036) and EPDS (*b* = 0.22, *p* = .015) scores, such that patients with elevated clinical outcomes across time had a longer treatment duration. However, this was not evident for BSI-GSI and PSOC scores (*p’s* > .114).

**Table 2:** Raw symptom scores for depression (EPDS), anxiety (STAI-T), and overall psychological distress (BSI-GSI), and parental sense of competence (PSOC) at admission, discharge, and 1-year follow-up among mothers with postpartum mental disorders (*N* = 348) and results from post-hoc comparisons between assessment points with FDR-corrected *p*-values.

	Admission (T0)		Discharge (T1)		1-year follow-up (T2)				Pairwise comparisons^a^				
	*M* (*SD*)	% above cut-off	*M* (*SD*)	% above cut-off	*M* (*SD*)	% above cut-off	ICC			*b*	*SE(b)*	*t*	*P_FDR_*
**EPDS**	15.82 (5.9)	83.7	9.76 (4.9)	50.0	8.92 (5.4)	44.3	.17		T0 – T1	**6.15**	**0.34**	**18.02**	**< .001**
	*(n=* 331)		*(n=* 306)		*(n=* 167)				T0 – T2	**6.59**	**0.42**	**15.56**	**< .001**
									T1 – T2	0.44	0.43	1.02	.307

**STAI-T**	54.77 (10.2)	79.3	46.68 (10.0)	50.0	44.56 (11.8)	42.7	.34		T0 – T1	**8.55**	**0.659**	**14.39**	**< .001**
	*(n=* 338)		*(n=* 306)		*(n=* 164)				T0 – T2	**10.15**	**0.75**	**13.55**	**< .001**
									T1 – T2	***1.60***	***0.76***	***2.11***	***.035***^b^

**BSI-GSI**	1.26 (0.67)	82.6	0.71 (0.49)	50.5	0.66 (0.55)	41.3	.32		T0 – T1	**0.56**	**0.03**	**17.25**	**< .001**
	*(n=* 339)		*(n=* 306)		*(n=* 167)				T0 – T2	**0.58**	**0.04**	**14.41**	**< .001**
									T1 – T2	0.03	0.04	0.65	.516

**PSOC**	59.03 (11.9)	N/A	67.28 (10.0)	N/A	67.72 (10.4)	N/A	.38		T0 – T1	**−8.59**	**0.61**	**−14.08**	**< .001**
	*(n=* 322)		*(n=* 298)		*(n=* 164)				T0 – T2	**−8.59**	**0.76**	**−11.28**	**< .001**
									T1 – T2	0.00	0.77	0.00	.999

*Note*. A positive *b* means that at Time 0 the score was higher than at Time 1 (e.g., T0 – T1). A negative *b* means that at Time 0 the score was lower than at Time 1 (e.g., T0 – T1). EPDS = Edinburgh Postnatal Depression Scale. STAI-*T* = State-Trait Anxiety Inventory – Trait Subscale. BSI-GSI = Brief Symptom Inventory - Global Severity Index. PSOC = Parenting Sense of Competence Scale. ICC = Intraclass Correlation Coefficient from an empty means linear mixed model. T0 = Admission. T1 = Discharge. T2 = 1-year follow-up.

a Results are based on linear mixed model with time and treatment duration as a fixed effect.

b This effect was not significant using multiple imputed data, *p* = .245.

RCI calculations showed that the minimum difference between two assessment points on our outcome measures required to be 95 % certain that changes were not solely due to measurement error were as follows: EPDS = 5.90; STAI = 6.92; BSI-GSI = 0.37; PSOC = 8.73 (see supplementary materials E).

### The effect of diagnostic group on treatment course

Diagnostic group showed a significant effect for all outcome measures with FDR-corrected pairwise comparisons (see Table 3) revealing that women with personality disorders had elevated scores on EPDS (*b’s* = −2.04 – 3.43, *p*’s < .007) and BSI-GSI (*b’s* = −0.23 – 0.48, *p*’s < .005) averaged across time compared to all other diagnostic groups. Women with personality disorders also had elevated STAI-T scores compared to women with schizophrenia/bipolar disorders (*b* = 6.97, *p* = .017) and in sensitivity analyses with multiple imputed data this was also the case for women with anxiety disorders (*b* = −3.55, *p* = .045; see supplementary materials D). Regarding PSOC levels, women with depressive disorders had significantly lower scores than women with anxiety disorders (*b* = −3.12, *p* = .030) and with schizophrenia/bipolar disorders (*b* = −6.47, *p* = .030), with the latter being at trend-level significant in sensitivity analyses using multiple imputed data (*b* = −4.88, *p* = .069; see supplementary materials D). Detailed information on estimated marginal means of each diagnostic group at admission, discharge, and 1-year follow-up and averaged across all time points is provided in the supplementary materials C.

**Table 3:** Results from post-hoc comparisons regarding clinical outcomes averaged across admission, discharge and 1-year follow-up between diagnostic groups with FDR-corrected p-values (*N* = 319).

	*b*	*SE (b)*	*t*	*P_FDR_*
**EPDS**				
Dep – Anx	0.06	0.57	0.11	.910
Dep – Pers	**−2.04**	**0.64**	**−3.18**	**.007**
Dep – Sch/Bip	1.39	1.10	1.26	.290
Anx – Pers	**−2.11**	**0.68**	**−3.08**	**.007**
Anx – Sch/Bip	1.32	1.13	1.17	.290
Pers – Schiz/Bip	**3.43**	**1.16**	**2.96**	**.007**

**STAI-T**				
Dep – Anx	0.43	1.14	0.37	.709
Dep – Pers	−2.64	1.28	−2.07	.074
Dep – Sch/Bip	4.33	2.19	1.97	.074
Anx – Pers	*−3.07*	*1.36*	*−2.25*	*.074*^a^
Anx – Sch/Bip	3.90	2.24	1.74	.099
Pers – Schiz/Bip	**6.97**	**2.31**	**3.02**	**.017**

**BSI-GSI**				
Dep – Anx	−0.08	0.06	−1.26	.210
Dep – Pers	**−0.32**	**0.07**	**−4.34**	**< .001**
Dep – Sch/Bip	0.17	0.13	1.35	.210
Anx – Pers	**−0.23**	**0.08**	**−3.03**	**.005**
Anx – Sch/Bip	0.25	0.13	1.96	.077
Pers – Schiz/Bip	**0.48**	**0.13**	**3.68**	**<.001**

**PSOC**				
Dep – Anx	**−3.21**	**1.23**	**−2.60**	**.030**
Dep – Pers	−1.06	1.39	−0.77	.444
Dep – Sch/Bip	***−6.47***	***2.42***	***−2.68***	***.030***^b^
Anx – Pers	2.14	1.48	1.45	.221
Anx – Sch/Bip	−3.27	2.47	−1.32	.225
Pers – Schiz/Bip	−5.41	2.55	−2.13	.069

*Note*. Results in italics indicate diverging findings when using multiply imputed (MI) data. A positive *b* means that Group 1 had a higher score than Group 2 (Group 1 – Group 2). A negative *b* means that Group 1 had a lower score than Group 2 (Group 1 – Group 2). Dep = Depressive disorders. Anx = Anxiety disorders/OCD/PTSD. Pers = Personality disorders. Sch/Bip = Schizophrenia/Bipolar disorders. EPDS = Edinburgh Postnatal Depression Scale. STAI-*T* = State-Trait Anxiety Inventory – Trait Subscale. BSI-GSI = Brief Symptom Inventory - Global Severity Index. PSOC = Parenting Sense of Competence Scale.

a using multiple imputed data, *p* = .040.

b using multiple imputed data, *p* = .069.

**Fig. 1:**
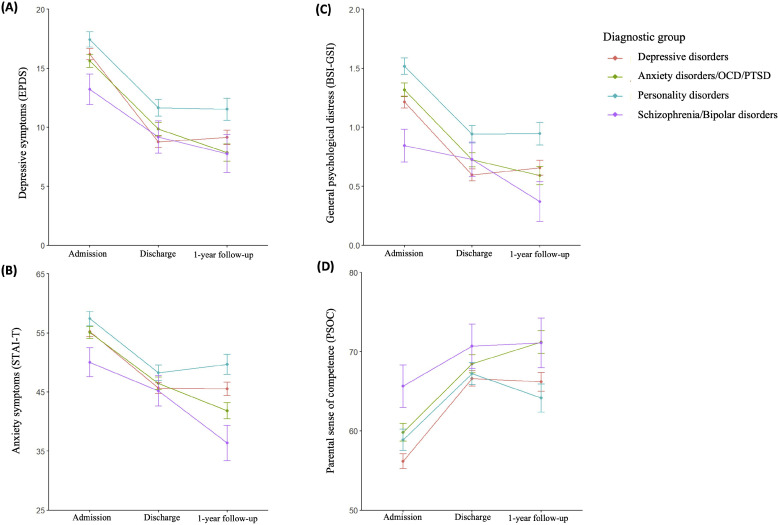
Line graph showing estimated mean clinical outcome scores at admission, discharge, and 1-year follow-up grouped by diagnostic group with standard error bars (*N=* 319). *Note.* Subsample sizes varied: depressive disorders (*n* = 66 – 132), anxiety disorders/OCD/PTSD (*n* = 45 – 95), personality disorders (*n* = 20 – 68), schizophrenia/bipolar disorders (*n* = 10 – 17). EPDS = Edinburgh Postnatal Depression Scale. STAI-*T* = State-Trait Anxiety Inventory – Trait Subscale. BSI-GSI = Brief Symptom Inventory - Global Severity Index. PSOC = Parenting Sense of Competence Scale. OCD = Obsessive compulsive disorder. PTSD = Posttraumatic Stress Disorder.

When the group by time interaction was added, FDR-corrected post-hoc interaction contrasts showed significant effects regarding STAI-T and BSI-GSI, but not EPDS or PSOC (see Fig. 1 and Table 4). Regarding BSI-GSI scores from admission to discharge, women with depressive disorders (*b* = −0.50, *p* = .007), anxiety disorders (*b* = −0.48, *p* = .007), and personality disorders (*b* = −0.46, *p* = .010) improved significantly more than women with schizophrenia/bipolar disorders. Regarding STAI-T scores from discharge to 1-year follow-up, patients with schizophrenia/bipolar disorders improved significantly more than patients with depressive disorders (*b* = 8.71, *p* = .020) and patients with personality disorders (*b* = 10.28, *p* = .020). Also, regarding STAI-T scores from discharge to 1-year follow-up, patients with anxiety disorders improved significantly more than patients with depressive disorders (*b* = 4.48, *p* = .023) and patients with personality disorders (*b* = −6.05, *p* = .020), yet this was not significant in sensitivity analyses using multiple imputed data (*bs* = −3.93 – 2.94, *p’s* = .189, see supplementary materials D).

**Table 4:** Results from post-hoc comparisons of change from admission to discharge and discharge to 1-year follow-up between diagnostic groups with FDR-corrected p-values (*N* = 330).

	Admission to discharge					Discharge to 1-year follow-up			
	*b*	*SE (b)*	*t*	*p*		*b*	*SE (b)*	*t*	*p*
**EPDS**									
Dep – Anx	−1.66	0.83	−2.00	.139		2.36	1.03	2.30	133
Dep – Pers	−1.65	0.95	−1.74	.167		0.50	1.21	0.42	.770
Dep – Sch/Bip	−3.37	1.59	−2.12	.139		1.79	1.91	0.93	.701
Anx – Pers	−0.01	1.00	0.01	.990		−1.86	1.29	−1.45	.447
Anx – Sch/Bip	−1.71	1.62	−1.06	.368		−0.58	1.96	−0.29	.770
Pers – Schiz/Bip	−1.73	1.69	−1.02	.368		1.29	2.06	0.62	.770

**STAI-T**									
Dep – Anx	−0.95	1.44	−0.66	.768		***4.48***	***1.83***	***2.44***	***.023***^a^
Dep – Pers	−0.39	1.63	−0.24	.810		−1.57	2.13	−0.74	.462
Dep – Sch/Bip	−4.76	2.77	−1.72	.357		**8.71**	**3.37**	**2.59**	**.020**
Anx – Pers	0.55	1.73	0.32	.810		***−6.05***	***2.28***	***−2.66***	***.020***^b^
Anx – Sch/Bip	−3.82	2.83	−1.35	.357		4.24	3.46	1.22	.266
Pers – Schiz/Bip	−4.37	2.93	−1.49	.357		**10.28**	**3.63**	**2.83**	**.020**

**BSI-GSI**									
Dep – Anx	−0.03	0.08	−0.32	.860		0.19	0.10	1.94	.144
Dep – Pers	−0.04	0.09	−0.47	.860		0.06	0.12	0.48	.632
Dep – Sch/Bip	**−0.50**	**0.15**	**−3.28**	**.007**		0.42	0.19	2.25	.144
Anx – Pers	−0.02	0.10	−0.18	.860		−0.14	0.13	−1.10	.325
Anx – Sch/Bip	**−0.48**	**0.16**	**−3.05**	**.007**		0.22	0.19	1.18	.325
Pers – Schiz/Bip	**−0.46**	**0.16**	**−2.84**	**.010**		0.36	0.20	1.80	.144

**PSOC**									
Dep – Anx	1.81	147	1.23	.328		−3.17	1.85	−1.71	.264
Dep – Pers	2.11	1.70	1.24	.328		2.64	2.17	1.22	.447
Dep – Sch/Bip	5.41	2.86	1.89	.328		−0.84	3.42	−0.25	.805
Anx – Pers	0.30	1.80	0.16	.870		5.81	2.31	2.52	.073
Anx – Sch/Bip	3.60	2.92	1.23	.328		2.33	3.51	0.66	.609
Pers – Schiz/Bip	3.30	3.04	1.09	.334		−3.49	3.68	−0.95	.517

*Note*. A negative *b* means that Group 1 had a greater reduction than Group 2 for the respective time point (Group 1 – Group 2). A positive *b* means that Group 2 had a greater reduction than Group 1 (Group 1 – Group 2). Dep = Depressive disorders. Anx = Anxiety disorders/OCD/PTSD. Pers = Personality disorders. Sch/Bip = Schizophrenia/Bipolar disorders. EPDS = Edinburgh Postnatal Depression Scale. STAI-*T* = State-Trait Anxiety Inventory – Trait Subscale. BSI-GSI = Brief Symptom Inventory - Global Severity Index. PSOC = Parenting Sense of Competence Scale.

a using multiple imputed data, *p* = .189.

b using multiple imputed data, *p* = .189.

### Associations with child behavioural problems at 1-year follow-up

Multiple linear regression analyses were conducted to examine whether CBCL scores in children at 1-year follow-up were predicted by changes in maternal clinical variables from admission to discharge and admission to 1-year follow-up, respectively. For analyses examining the effect of symptom change from admission to discharge, hierarchical regressions with pre-treatment symptomatology, treatment duration, and change score were added to Model 1. Model 2 added maternal clinical symptom severity of the respective outcome at 1-year follow-up. Findings revealed a significant association between improvements in clinical outcomes from admission to discharge with CBCL scores at 1-year follow-up for EPDS and BSI-GSI (*p*’s < .033), but not for STAI-T or PSOC (*p*’s > .135). However, while the effect for EPDS was not significant in sensitivity analyses using multiple imputed data (*p* = .258), the effect for BSI-GSI was trend-level significant (*p* = .076; see supplementary materials D). Adding maternal clinical symptoms at 1-year follow-up in model 2 rendered this effect non-significant and contributed to an additional explanation of 8–12 % of variance (*p*’s < .001; see Table 5).

**Table 5:** Results of multiple regression analyses predicting overall child behaviour problems (CBCL) from maternal symptom change across treatment and until 1-year follow-up.

	β	*B*	*SE*	*p*	*R^2^*
**Depressive symptoms (EPDS)**					
**Admission to discharge**					
*Model 1*^a^:					.04
Admission to discharge EPDS	***−.26***	***−.68***	***0.31***	***.033***^b^	
*Model 2*^a^:					.13
Admission to discharge EPDS	−.12	−.31	0.32	.334	
1-year follow-up EPDS	**.34**	**0.94**	**0.24**	**<.001**	
					
**Admission to 1-year-follow-up**					
*Model 1*^a^:					.13
Admission to 1-year-follow-up EPDS	**−.45**	**−1.02**	**0.22**	**< .001**	
					
**Anxiety symptoms (STAI-T)**					
**Admission to discharge**					
*Model 1*^a^:					.03
Admission to discharge STAI-T	−.14	−.22	0.15	.135	
*Model 2*^a^:					.15
Admission to discharge STAI-T	.01	0.02	0.15	.915	
1-year follow-up STAI-T	**.43**	**0.54**	**0.12**	**<.001**	
					
**Admission to 1-year follow-up**					
*Model 1*^a^:					.17
Admission to 1-year follow-up STAI-T	**−.42**	**−.54**	**0.11**	**< .001**	
					
**General psychological distress (BSI-GSI)**					
**Admission to discharge**					
*Model 1*^a^:					.07
Admission to discharge BSI-GSI	***−.32***	***−8.63***	***3.11***	***.006***^c^	
*Model 2*^a^:					.19
Admission to discharge BSI-GSI	−.07	−1.85	3.24	.569	
1-year follow-up BSI-GSI	**.47**	**12.83**	**2.72**	**<.001**	
					
**Admission to 1-year follow-up**					
*Model 1*^a^:					.18
Admission to 1-year follow-up BSI-GSI	**−.54**	**−12.32**	**2.20**	**< .001**	
					
**Parental sense of competence (PSOC)**					
**Admission to discharge**					
*Model 1*^a^:					.06
Admission to discharge PSOC	-.12	-0.18	0.16	.254	
*Model 2*^a^:					.14
Admission to discharge PSOC	.04	.06	0.16	.714	
1-year follow-up PSOC	**−.35**	**−.50**	**0.14**	**<.001**	
					
**Admission to 1-year follow-up**					
*Model 1*^a^:					.15
Admission to 1-year follow-up PSOC	**−.38**	**−.48**	**0.12**	**< .001**	

*Note*. Change scores from admission to discharge and admission to 1-year follow-up were calculated such that a negative regression coefficient signifies that a greater improvement in maternal symptoms was associated with lower CBCL scores.

a controlled for the respective measure at admission and treatment duration. CBCL = Childs Behaviour Checklist, EPDS = Edinburgh Postnatal Depression Scale. STAI-*T* = State-Trait Anxiety Inventory – Trait Subscale. BSI-GSI = Brief Symptom Inventory - Global Severity Index. PSOC = Parenting Sense of Competence Scale.

b using multiple imputed data, *p* = .258.

c using multiple imputed data, *p* = .076.

**Fig. 2:**
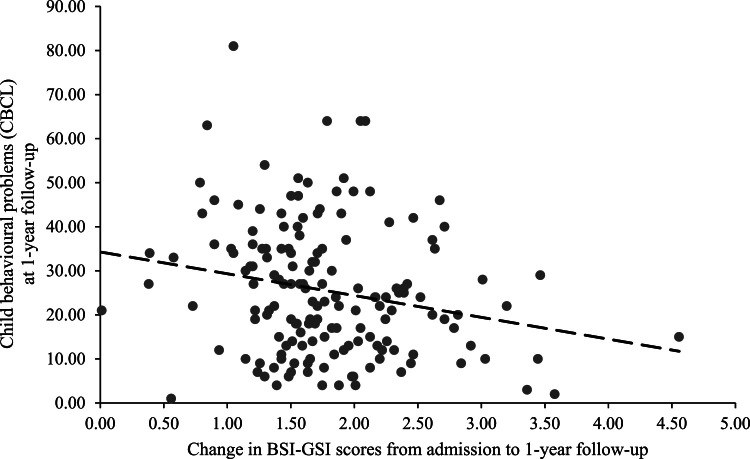
Scatterplot with a linear trendline showing the association between maternal change in overall psychological distress (BSI-GSI) from admission to 1-year follow-up and child behavioural problems (CBCL) at 1-year follow-up. *Note.* A constant of 1.20 was added to the change score in BSI-GSI from admission to 1-year follow-up for visualisation purposes. Linear regression results showed a significant association between the change in BSI-GSI from admission to 1-year follow-up and child behavioural outcomes at 1-year follow-up, controlling for pre-treatment symptom level as well as treatment duration (β = −0.54, *p* < .001). BSI-GSI = Brief Symptom Inventory - Global Severity Index. CBCL = Child Behaviour Checklist.

Regarding symptom change from admission to 1-year follow-up, controlling for pre-treatment symptomatology and treatment duration, greater maternal improvement in the EPDS, STAI-T, BSI-GSI, and PSOC was associated with lower CBCL scores in their children assessed at 1-year follow-up (see Table 5 for regression results and Fig. 2 for the effect with BSI-GSI). The pattern of results was confirmed for both externalizing and internalizing subscale symptoms except that the effect of EPDS change from admission to discharge was no longer significant in model 1 for either internalizing nor externalizing symptoms (data not shown).

## DISCUSSION

The aim of this study was to evaluate the potential benefits of an interaction-focused treatment at a MBU day clinic on maternal psychological and parenting outcomes up to 1-year follow-up. Our findings revealed statistically significant improvements across psychopathological outcome measures from admission to discharge which were also clinically significant, i.e., reliable and reflecting change above fluctuations due to imprecise measurement. Results indicated that only anxiety symptoms showed a small improvement from discharge to 1-year follow-up, albeit this was not confirmed in sensitivity analyses with multiple imputed data. Hence, our results indicate that clinical outcomes largely remained stable after discharge. However, given the absence of a waiting list control group, these findings should be interpreted cautiously, as no conclusions can be drawn to what extend the dyadic treatment programme contributed to the observed improvements. Additional analyses indicated that maternal primary clinical diagnosis contributed to differences in the severity of all outcome measures as well as to different outcome trajectories regarding anxiety symptoms and overall psychological distress. Treatment may also have benefited the offspring, as greater maternal symptom improvement from admission to 1-year follow-up was associated with fewer child behavioural problems at 1-year follow-up. However, this effect was not found for the clinical symptom change from admission to discharge when controlling for current maternal clinical symptoms at 1-year follow-up.

Previous studies have shown immediate beneficial effects of a dyadic mother-child treatment on maternal psychopathology and mother-child bonding in an inpatient (Christl et al., 2015; reviewed in Connellan et al., 2017; Glangeaud-Freudenthal et al., 2011; Salmon et al., 2003) and day clinic setting (Weidner et al., 2021). However, available data regarding the stability of these effects over longer time periods are limited. The current study revealed that, among women with postpartum mental disorders, improvements of maternal psychological symptoms (i.e., depressiveness, anxiety, overall psychological distress) and parenting outcomes remained stable up to one year after discharge. These findings tentatively indicate that treatment-associated effects might be maintained long-term, highlighting the potential of combining life-stage-specific and individual disorder-specific therapy components for long-term psychotherapeutic outcome. Further, our data lend support to the notion that focusing on the promotion of parenting competence in a sensitive time window can lead to changes in mothers’ behavioural strategies and attitudes that persist in the long-term. However, due to the lack of a waiting list control group, these findings should be interpreted cautiously since we cannot rule out confounding effects that may occur over time such as spontaneous remission, the natural process of adaptation to motherhood, or general improvements in parenting skills. Hence, although clinically relevant changes were detected, it remains unclear to what extent the dyadic treatment programme contributed to the observed effects in clinical changes.

In line with previous findings on a day clinic treatment response from admission to discharge (Weidner et al., 2021), the current data showed an association between primary clinical diagnosis and the severity of psychological outcomes averaged across time. There was also an effect of clinical diagnosis on the long-term course of clinical outcomes regarding anxiety and overall psychological distress. However, no consistent pattern was found across measures, and the few differences between diagnostic groups were overall small and therefore should be interpreted cautiously in terms of their clinical significance. Specifically, women with personality disorders exhibited higher symptom scores averaged across all time points for depressive symptoms, overall psychological distress and (in part) anxiety compared to the other diagnostic groups. Considering that personality disorders (mainly borderline personality disorder in our sample) are a chronic disorder characterized by severe affective instability and poor interpersonal functioning (e.g., Gunderson et al., 2018), it is conceivable that affected women might show high levels of stress and psychopathology in the postpartum period as it is associated with multiple unfamiliar and challenging situations (for borderline personality disorders reviewed in Florange & Herpertz, 2019). In addition, women with personality disorders had the most unfavourable sociodemographic characteristics (e.g., higher number of children, lower educational status, highest percentage of single mothers) compared to the other diagnostic groups, likely contributing to their increased psychopathological burden. Despite these challenges, this group’s average reported parental sense of competence across all time points was not significantly lower. However, at 1-year follow-up, our data indicated that these mothers had the lowest descriptive values, with significant differences as compared to mothers with anxiety disorders. This fits with evidence suggesting mothers with (borderline) personality disorders frequently exhibit dysfunctional parenting behaviours such as insensitivity, overprotection, and hostility (Eyden et al., 2016), which may persist more even after day clinic treatment, thus highlighting the need for continued and targeted support for mothers with personality disorders regarding parenting competence.

The heightened psychopathological symptom burden in this diagnostic group underscores the relevance of parent-specific treatment programs tailored to the unique needs of patients with (borderline) personality disorders during the postpartum period (e.g., Buck-Horstkotte et al., 2022; Williams et al., 2021). Such programs can particularly improve women’s emotional regulation, addressing uncertainties about their child's cues and low self-esteem, which in turn may improve women's mentalisation capability (Francis et al., 2023). Addressing dysfunctional parenting behavioural patterns is also essential to allow the establishment of a healthy mother-child relationship and to support the emotional development of offspring (Florange & Herpertz, 2019). This is of particular relevance considering that offspring of mothers with borderline personality disorders are at increased risk of showing more adverse clinical outcomes, including insecure attachment patterns, emotional dysregulation, and various expressions of psychopathology (e.g., internalising and externalising problems, borderline personality disorder symptoms, Eyden et al., 2016).

Besides the increased psychopathological symptom burden in mothers with personality disorders over time, our findings also revealed a differential symptom trajectory in anxiety symptoms. Specifically, mothers with personality disorders showed a slight worsening in anxiety levels from discharge to 1-year follow-up, which contrasted significantly with the improvements observed for mothers with schizophrenia/bipolar disorders and anxiety disorders, albeit the latter contrast was not confirmed by sensitivity analyses using multiple imputed data. This finding emphasizes that ongoing, closely integrated psychotherapeutic support following day clinic treatment may be especially important for mothers with personality disorders. A parent-specific treatment programme should extend into the outpatient setting to foster coherent mother-child interactions and emotion regulation among affected mothers to reduce anxiety related to motherhood and strengthen satisfaction in the parenting role. Given our descriptive results of a lower socioeconomic status of mothers with personality disorders compared to the other diagnostic groups, these mothers may also benefit significantly from additional social therapeutic services.

Our results further indicated that mothers with anxiety disorders had significantly higher parental sense of competence at 1-year follow-up than mothers with personality disorders and averaged across time than mothers with depressive disorders. Moreover, results indicated that mothers with anxiety disorders improved significantly more in anxiety symptoms from discharge to 1-year follow-up than mothers with personality and depressive disorders, although this was not significant in sensitivity analyses using multiple imputed data. The specific pattern of a tentative improvement in anxiety symptoms among mothers with a primary diagnosis of anxiety disorders is intriguing and we can only speculate about possible reasons. Most mothers with anxiety disorders report anxious thoughts and feelings related to their infant, accompanied by avoidance behaviour. These symptoms are primarily targeted by individual psychotherapeutic treatment sessions mainly through exposure-based treatment. It is conceivable that the treatment effects of mothers with anxiety disorders due to exposure-based therapy may result in a further improved handling of child-related fear of the mothers to the baby in the post-treatment period. Additionally, it is conceivable that anxious thoughts and feelings about the infant may simply decrease because the child gets older and, from a subjective perspective, becomes less vulnerable, and thus parental self-efficacy may naturally increase over time. However, due to a lacking waiting list control group of mothers with anxiety disorders, this alternative explanation remains speculative and cannot be ruled out. Another possible explanation is that mothers with anxiety disorders may have benefited more from outpatient treatment services post-discharge compared to those with personality disorders and depressive disorders. However, analyses revealed that 91.9 % of women reported continuing treatment or receiving assistance after discharge, regardless of their diagnostic status.

Mothers with a primary diagnosis of schizophrenia/bipolar disorders showed a marked improvement in anxiety symptoms from discharge to 1-year follow-up, in contrast to those with personality disorders and depressive disorders. This further improvement in anxiety symptoms may be attributed to continued stabilization, adaptation to treatment, and development of better coping strategies over time. Descriptive inspection of the severity of outcomes suggests that this group had the lowest pre-treatment symptom load as compared to the other diagnostic groups, with significant differences compared to mothers with personality disorders (depression, anxiety, and overall general distress), depressive disorders (overall general distress, parental sense of competence), as well as anxiety disorders (overall general distress). This may also explain the finding that affected mothers exhibited a diminished reduction in symptoms of general psychological distress from admission to discharge, as compared to all other diagnostic groups, potentially reflecting a floor effect. It is important to note that most of affected mothers experienced symptoms particularly during the puerperium and were often treated in a psychiatric unit (including psychopharmacological treatment) prior to their admission to our MBU. Hence, it is plausible that these patients may have already benefited regarding symptom improvement before MBU admission. Also, it should be considered that the schizophrenia/bipolar disorder subsample was the smallest diagnostic group in this study, comprising only *n* = 17 individuals, and thus effects should be interpreted cautiously.

Regarding mothers with depressive disorders, our data showed that this group exhibited significantly lower levels in parental sense of competence across time as compared to mothers with anxiety disorders and tentatively to schizophrenia/bipolar disorders, while no differences were found relative to personality disorders. This finding points towards the potential value of providing ongoing and targeted psychotherapeutic support to foster parenting competence among mothers with depressive disorders. Interestingly, a prior study revealed that mothers with depressive disorders showed the greatest improvements from admission to discharge regarding maternal-child-bonding compared to the other diagnostic groups (Weidner et al., 2021). Similarly, our descriptive data on psychopathology measures indicate that mothers with depressive disorders showed a marked improvement from admission to discharge, although this effect was only significant regarding overall psychological distress compared to women with schizophrenia/bipolar disorders. Interestingly, despite this short-term improvement, these mothers showed a slight worsening on average from discharge to 1-year follow-up, with significant effects regarding levels of anxiety when compared to mothers with schizophrenia/bipolar disorders and mothers with anxiety disorders. This might fit with meta-analytical findings based on RCT studies from outpatient settings suggesting that mothers with postpartum depression do not benefit from dyadic psychotherapy at later follow-up assessments (Huang et al., 2020). It is conceivable that the positive effects offered by an intensive day clinic setting, for example in terms of daily structure, cognitive restructuring (particularly addressing feelings of insufficiency), and addressing the bonding disorder may not be fully maintained for these women through outpatient care post-discharge. In addition, the symptomatology of postpartum major depression is often linked to stable self-esteem issues triggered by the transition to motherhood. These issues, which extend beyond motherhood and affect various areas of life, may not be addressed and thus remain relevant at follow-up, hindering further clinical improvement. Together, our findings indicate that closer and more intensive follow-up care would be beneficial for mothers with depressive disorders to prevent a possible deterioration in clinical outcomes (mainly anxiety levels) after discharge.

Another objective of the current study was to investigate whether clinical symptom change after an interaction-focused MBU treatment corresponded with reduced subsequent child behavioural problems. Notably, at 1-year follow-up, only 3.7 % of the children were above the clinically relevant cut-off for overall behavioural problems. Compared to prevalence rates of emotional and behavioural problems in German samples (Barkmann & Schulte-Markwort, 2005), this indicates no higher prevalence among children of mothers with postpartum mental disorders treated in our day clinic MBU. Importantly, after controlling for initial symptom severity and treatment duration, greater maternal symptom improvement from admission to 1-year follow-up was related to fewer behavioural problems in their children. This was evident for all outcome measures with most pronounced effects for maternal general psychological distress. Interestingly, from admission to discharge this effect only emerged for depressive symptoms and overall psychological distress and disappeared when controlling for maternal clinical symptomatology at 1-year follow-up. Given that the consideration of maternal clinical symptomatology at 1-year follow-up contributed to an additional explanation of 8–12 % of variance, it is conceivable that current psychopathology may be even more important than clinical symptom changes from admission to discharge in predicting child behavioural problems. This highlights the importance of post-discharge maternal symptom development for child behavioural problems and speaks to the relevance of clinical follow-up care to prevent the development of child behavioural problems. Our findings add to previous research emphasizing the importance of targeting both maternal psychopathology as well as mother-baby interaction for child behavioural development (e.g., Aktar et al., 2019; Poobalan et al., 2007) and extend these findings to a day clinic setting. However, future research should confirm these findings considering a control group, include longer follow-up assessments and examine other development aspects (e.g., cognitive-, fine or gross motor development, communication) to closely evaluate the long-term impact on offspring development.

One of the main limitations of the current study is the lack of a waiting control group. Since therapy places are given depending on treatment urgency within a narrow window for intervention, such a study design was ethically unfeasible. This limitation has significant implications for the interpretation of the results. The absence of a control group precludes the possibility of ruling out the influence of factors unrelated to the intervention. These may include spontaneous remission, the natural process of adaptation to motherhood, or improvements in parenting skills over time that may occur independently of treatment. For instance, the transition to parenthood frequently involves substantial personal growth and the acquisition of new skills, which could contribute to the positive changes documented in the findings. Additionally, unmeasured variables like environmental or societal stressors may have affected the outcomes. In light of these considerations, a cautious interpretation of the findings is warranted, acknowledging that improvements in clinical outcomes may reflect contributions beyond the dyadic day clinic MBU treatment. Nevertheless, the clinically relevant improvements observed in the study indicate that treatment is likely to have played a substantial role, making it improbable that the observed changes could be attributed solely to these factors. With regards to spontaneous remission, meta-analytical findings suggested short-term remission rates in major depression ranging from 8 % to 18 % (Mekonen et al., 2022). As the current data revealed that 33.1 % of mothers fell below the threshold for clinically relevant depression after discharge, it can be assumed that expected rates of natural remission may have been exceeded in the current treatment sample.

Another limitation concerns the high drop-out rate of 52 % at 1-year follow-up. Following recommendations for dealing with missing data in clinical research, we calculated sensitivity analyses with multiple imputation (Austin et al., 2021). This approach provides less biased estimates and enables the inclusion of all participants’ data, thereby preventing bias by excluding them from analyses. Comparison of the results with and without multiple imputation showed minor changes which we acknowledge throughout, lending confidence to the robustness of the effects that were confirmed in both approaches. While this approach is appropriate for our data, future studies are still required to analyse the reasons for the high drop-out rate, potentially through the implementation of phone interviews or brief questionnaires. Furthermore, all outcome measures were based on maternal self-report. Since the evaluation of mother-child bonding and child behavioural problems may be related to maternal clinical symptomatology (e.g., Gartstein et al., 2009; Wesselhoeft et al., 2021), further studies are needed integrating data of partners and other close family members or evaluating mother-child interactions and child behavioural problems by trained staff. Similarly, the assessment of psychotherapeutic treatment outcome would benefit from evaluations by treatment personnel (e.g., physicians, psychotherapists, nurses). While our data underline the usefulness of a MBU day treatment for maternal psychopathological outcomes, it is noteworthy that approximately 50 % (at discharge) and 44 % (at 1-year follow-up) of mothers remained above clinically relevant cut-offs, indicating that they may not have benefited sufficiently from the intervention. This observation may indicate that postpartum mental disorders are particularly severe and/or persistent (Vliegen et al., 2010). Further research investigating bio-psycho-social mechanisms at multiple levels of measurement is needed to elucidate knowledge on factors that contribute to the efficacy of MBU treatment. Such an approach may increase our ability to alleviate further suffering for mothers, their partners, and their offspring as well as to develop and implement more tailored interventions (Bergunde et al., 2022).

## Conclusion

This study examined the potential benefits of an interaction-focused MBU day clinic treatment on maternal psychological and parenting outcomes up to 1-year follow-up. Significant and clinically meaningful improvements were observed in all outcome measures from admission to discharge. Notably, no additional symptom changes were seen from discharge to 1-year follow-up, except for anxiety symptoms, indicating that clinical improvements remained stable. However, the lack of a waiting list control group poses a critical limitation and does not allow to draw definitive conclusions regarding the efficacy of the MBU day clinic treatment.

In addition, the current findings suggest that clinical symptom burden regarding all measures and trajectories regarding anxiety and overall psychological distress varied depending on the main clinical diagnosis. Specifically, our findings underline the need for ongoing, intensive follow-up care for mothers, particularly those with personality disorders. Maternal symptom improvement from admission to 1-year follow-up was associated with fewer child behavioural problems at 1-year follow-up, indicating a potential transgenerational impact of the treatment. However, this relationship was not found for the clinical symptom change from admission to discharge when controlling for current maternal symptomatology at 1-year follow-up, underscoring the importance of maternal symptom development post-discharge for developing child behavioural problems.

## Funding

This research did not receive any specific grant from funding agencies in the public, commercial, or not-for-profit sectors.

### Declaration of generative AI and AI-assisted technologies in the writing process

During the preparation of this work Deepl and ChatGPT were used in order to improve the readability and language of the manuscript. After using this tool/service, the authors reviewed and edited the content as needed and take full responsibility for the content of the published article.

## Declaration of competing interest

KW receives third-party funding for the development of a S3 guideline on both peripartum mental disorders and peripartum trauma. The other authors have nothing to declare.

## Appendix Supplementary materials

Image, application 1

## References

[bib0001] Achenbach T., Rescorla L. (2000). Child behavior checklist for ages 1 1/2-5. Reporter.

[bib0002] Achenbach T.M. (1991).

[bib0003] Aktar E., Qu J., Lawrence P.J., Tollenaar M.S., Elzinga B.M., Bogels S.M. (2019). Fetal and infant outcomes in the offspring of parents with perinatal mental disorders: Earliest influences. Frontiers in psychiatry.

[bib0004] Austin P.C., White I.R., Lee D.G.S., van Buuren S. (2021). Missing data in clinical research: A tutorial on multiple imputation. Canadian Journal of Cardiology.

[bib0005] Bakermans-Kranenburg M.J., Van Ijzendoorn M.H., Juffer F. (2003). Less is more: Meta-analyses of sensitivity and attachment interventions in early childhood. Psychological bulletin.

[bib0006] Barkmann C., Schulte-Markwort M. (2005). Emotional and behavioral problems of children and adolescents in Germany–an epidemiological screening. Social psychiatry and psychiatric epidemiology.

[bib0007] Bauer A., Parsonage M., Knapp M., Iemmi V., Adelaja B., Hogg S. (2014).

[bib0008] Bergant A., Nguyen T., Moser R., Ulmer H. (1998). Prevalence of depressive disorders in early puerperium]. Gynakologisch-geburtshilfliche Rundschau.

[bib0009] Bergunde L., Garthus-Niegel S., Alexander N., Steudte-Schmiedgen S. (2022). Perinatal mental health research: Towards an integrative biopsychosocial approach. Taylor & Francis.

[bib0010] Bittner A., Coenen A., Garthus-Niegel S., Weidner K. (2021). Frühe bindungsfördernde Eltern-Kind-therapie bei psychischen störungen in der postpartalzeit. Ärztliche Psychotherapie.

[bib0011] Brockington I.F., Fraser C., Wilson D. (2006). The Postpartum Bonding Questionnaire: A validation. Archives of women's mental health.

[bib0012] Brockington I.F., Oates J., George S., Turner D., Vostanis P., Sullivan M., Loh C., Murdoch C. (2001). A screening questionnaire for mother-infant bonding disorders. Archives of women's mental health.

[bib0013] Buck-Horstkotte S., Renneberg B., Rosenbach C. (2022).

[bib0014] Christl B., Reilly N., Yin C., Austin M.-P. (2015). Clinical profile and outcomes of women admitted to a psychiatric mother-baby unit. Archives of women's mental health.

[bib0015] Connellan K., Bartholomaeus C., Due C., Riggs D.W. (2017). A systematic review of research on psychiatric mother-baby units. Archives of Women's Mental Health.

[bib0016] Cooper P.J., Murray L., Wilson A., Romaniuk H. (2003). Controlled trial of the short- and long-term effect of psychological treatment of post-partum depression. I. Impact on maternal mood. The British journal of psychiatry.

[bib0017] Cox J.L., Holden J.M., Sagovsky R. (1987). Detection of postnatal depression. Development of the 10-item Edinburgh Postnatal Depression scale. The British journal of psychiatry.

[bib0018] Dennis C.-L., Falah-Hassani K., Shiri R. (2017). Prevalence of antenatal and postnatal anxiety: Systematic review and meta-analysis. The British Journal of Psychiatry.

[bib0019] Derogatis L.R. (1993).

[bib0020] Erickson N., Julian M., Muzik M. (2019). Perinatal depression, PTSD, and trauma: Impact on mother-infant attachment and interventions to mitigate the transmission of risk. International review of psychiatry (Abingdon, England).

[bib0021] Eyden J., Winsper C., Wolke D., Broome M.R., MacCallum F. (2016). A systematic review of the parenting and outcomes experienced by offspring of mothers with borderline personality pathology: Potential mechanisms and clinical implications. Clinical Psychology Review.

[bib0022] Florange J.G., Herpertz S.C. (2019). Parenting in patients with borderline personality disorder, sequelae for the offspring and approaches to treatment and prevention. Current psychiatry reports.

[bib0023] Forman D.R., O'Hara M.W., Stuart S., Gorman L.L., Larsen K.E., Coy K.C. (2007). Effective treatment for postpartum depression is not sufficient to improve the developing mother-child relationship. Development and psychopathology.

[bib0024] Francis J.L., Sawyer A., Roberts R., Yelland C., Drioli-Phillips P., Williams A.E.S. (2023). Mothers with borderline personality disorders' experiences of mother-infant dialectical behavior therapy. Journal of Clinical Psychology.

[bib0025] Franke G.H. (2000).

[bib0026] Gartstein M.A., Bridgett D.J., Dishion T.J., Kaufman N.K. (2009). Depressed mood and maternal report of child behavior problems: Another look at the depression-distortion hypothesis. Journal of applied developmental psychology.

[bib0027] Glangeaud-Freudenthal N.-C., Sutter A.-L., Thieulin A.-C., Dagens-Lafont V., Zimmermann M.-A., Debourg A., Massari B., Cazas O., Cammas R., Rainelli C. (2011). Inpatient mother-and-child postpartum psychiatric care: Factors associated with improvement in maternal mental health. European psychiatry.

[bib0028] Göbel A., Lüersen L., Asselmann E., Arck P., Diemert A., Garthus-Niegel S., Mudra S., Martini J. (2024). Psychometric properties of the Maternal postnatal attachment Scale and the postpartum bonding Questionnaire in three German samples. Bmc Pregnancy and Childbirth.

[bib0029] Goodman J.H., Watson G.R., Stubbs B. (2016). Anxiety disorders in postpartum women: A systematic review and meta-analysis. Journal of Affective Disorders.

[bib0030] Graham J.W. (2009). Missing data analysis: Making it work in the real world. Annual Review of Psychology.

[bib0031] Guhn M., Forer B., Zumbo B.D., Michalos A.C. (2014). Encyclopedia of quality of life and well-being research.

[bib0032] Gunderson J.G., Herpertz S.C., Skodol A.E., Torgersen S., Zanarini M.C. (2018). Borderline personality disorder. Nature reviews. Disease primers.

[bib0033] Hahn-Holbrook J., Cornwell-Hinrichs T., Anaya I. (2018). Economic and health predictors of national postpartum depression prevalence: A systematic review, meta-analysis, and meta-regression of 291 studies from 56 countries. Frontiers in psychiatry.

[bib0034] Heyne C.-S., Kazmierczak M., Souday R., Horesh D., Lambregtse-van den Berg M., Weigl T., Horsch A., Oosterman M., Dikmen-Yildiz P., Garthus-Niegel S. (2022). Prevalence and risk factors of birth-related posttraumatic stress among parents: A comparative systematic review and meta-analysis. Clinical psychology review.

[bib0035] Hoffman L. (2015). Longitudinal analysis: Modeling within-person fluctuation and change. Longitudinal Analysis: Modeling within-Person Fluctuation and Change.

[bib0036] Howard L.M., Khalifeh H. (2020). Perinatal mental health: A review of progress and challenges. World psychiatry : Official journal of the World Psychiatric Association (WPA).

[bib0037] Huang R., Yang D., Lei B., Yan C., Tian Y., Huang X., Lei J. (2020). The short- and long-term effectiveness of mother-infant psychotherapy on postpartum depression: A systematic review and meta-analysis. Journal of affective disorders.

[bib0038] Jacobson N.S., Truax P. (1991). Clinical-significance - a statistical approach to defining meaningful change in psychotherapy-research. Journal of Consulting and Clinical Psychology.

[bib0039] Johnston C., Mash E.J. (1989). A measure of parenting satisfaction and efficacy. Journal of Clinical Child Psychology.

[bib0040] Kersten-Alvarez L.E., Hosman C.M., Riksen-Walraven J.M., Van Doesum K.T., Hoefnagels C. (2010). Long-term effects of a home-visiting intervention for depressed mothers and their infants. Journal of child psychology and psychiatry, and allied disciplines.

[bib0041] Laux L. (1981).

[bib0042] Liu X., Wang S., Wang G. (2022). Prevalence and risk factors of postpartum depression in women: A systematic review and meta-analysis. Journal of Clinical Nursing.

[bib0043] Marcus S.M. (2009). Depression during pregnancy: Rates, risks and consequences. Journal of Population Therapeutics and Clinical Pharmacology.

[bib0044] Mascheroni E., Ionio C. (2019). The efficacy of interventions aimed at improving post-partum bonding: A review of interventions addressing parent-infant bonding in healthy and at risk populations. Journal of Neonatal Nursing.

[bib0045] Mekonen T., Ford S., Chan G.C.K., Hides L., Connor J.P., Leung J. (2022). What is the short-term remission rate for people with untreated depression? A systematic review and meta-analysis. Journal of affective disorders.

[bib0046] Mountain G., Cahill J., Thorpe H. (2017). Sensitivity and attachment interventions in early childhood: A systematic review and meta-analysis. Infant Behavior and Development.

[bib0047] Murray L., Cooper P.J., Wilson A., Romaniuk H. (2003). Controlled trial of the short- and long-term effect of psychological treatment of post-partum depression: 2. Impact on the mother-child relationship and child outcome. The British journal of psychiatry.

[bib0048] Netsi E., Pearson R.M., Murray L., Cooper P., Craske M.G., Stein A. (2018). Association of persistent and severe postnatal depression with child outcomes. JAMA psychiatry.

[bib0049] Newman D.A. (2014). Missing data: Five practical guidelines. Organizational Research Methods.

[bib0050] Nylen K.J., Moran T.E., Franklin C.L., O'Hara M W. (2006). Maternal depression: A review of relevant treatment approaches for mothers and infants. Infant mental health journal.

[bib0051] Ohan J.L., Leung D.W., Johnston C. (2000). The parenting sense of competence scale: Evidence of a stable factor structure and validity. Canadian Journal of Behavioural Science.

[bib0052] Organization, W.H. (2004).

[bib0053] Pierce M., Hope H.F., Kolade A., Gellatly J., Osam C.S., Perchard R., Kosidou K., Dalman C., Morgan V., Di Prinzio P., Abel K.M. (2020). Effects of parental mental illness on children's physical health: Systematic review and meta-analysis. The British journal of psychiatry.

[bib0054] Poobalan A.S., Aucott L.S., Ross L., Smith W.C., Helms P.J., Williams J.H. (2007). Effects of treating postnatal depression on mother-infant interaction and child development: Systematic review. The British journal of psychiatry.

[bib0055] Prasad D., Kuhathasan N., de Azevedo Cardoso T., Suh J.S., Frey B.N. (2022). The prevalence of borderline personality features and borderline personality disorder during the perinatal period: A systematic review and meta-analysis. Archives of women's mental health.

[bib0056] Reck C., Klier C.M., Pabst K., Stehle E., Steffenelli U., Struben K., Backenstrass M. (2006). The German version of the Postpartum Bonding Instrument: Psychometric properties and association with postpartum depression. Archives of women's mental health.

[bib0057] Salmon M., Abel K., Cordingley L., Friedman T., Appleby L. (2003). Clinical and parenting skills outcomes following joint mother–baby psychiatric admission. Australian & New Zealand Journal of Psychiatry.

[bib0058] Sharpe D., Cribbie R.A. (2023). Analysis of treatment-control pre-post-follow-up design data. Quantitative Methods for Psychology.

[bib0059] Shorey S., Chee C.Y.I., Ng E.D., Chan Y.H., San Tam W.W., Chong Y.S (2018). Prevalence and incidence of postpartum depression among healthy mothers: A systematic review and meta-analysis. Journal of psychiatric research.

[bib0060] Spielberger C.D., Gorsuch R., Lushene R., Vagg P., Jacobs G. (1970). Manual for the state-trait inventory. Consulting Psychologists, Palo Alto, California.

[bib0061] Stein A., Netsi E., Lawrence P.J., Granger C., Kempton C., Craske M.G., Nickless A., Mollison J., Stewart D.A., Rapa E., West V., Scerif G., Cooper P.J., Murray L. (2018). Mitigating the effect of persistent postnatal depression on child outcomes through an intervention to treat depression and improve parenting: A randomised controlled trial. The lancet. Psychiatry.

[bib0062] Stein A., Pearson R.M., Goodman S.H., Rapa E., Rahman A., McCallum M., Howard L.M., Pariante C.M. (2014). Effects of perinatal mental disorders on the fetus and child. Lancet (London, England).

[bib0063] Team, R.C. (2023).

[bib0064] Tsivos Z.-L., Calam R., Sanders M.R., Wittkowski A. (2015). Interventions for postnatal depression assessing the mother–infant relationship and child developmental outcomes: A systematic review. International journal of women's health.

[bib0065] van Buuren S., Groothuis-Oudshoorn K. (2011). mice: Multivariate imputation by chained equations in R. Journal of Statistical Software.

[bib0066] Vesga-Lopez O., Blanco C., Keyes K., Olfson M., Grant B.F., Hasin D.S. (2008). Psychiatric disorders in pregnant and postpartum women in the United States. Archives of general psychiatry.

[bib0067] Vliegen N., Luyten P., Besser A., Casalin S., Kempke S., Tang E. (2010). Stability and change in levels of depression and personality: A follow-up study of postpartum depressed mothers that were hospitalized in a mother-infant unit. The Journal of nervous and mental disease.

[bib0068] Weidner K., Bergunde L., Frohberg J., Coenen A., Steudte-Schmiedgen S. (2023). Women’s health and pandemic crisis.

[bib0069] Weidner K., Junge-Hoffmeister J., Coenen A., Croy I., Bittner A. (2021). Verbesserung der psychischen Gesundheit und bindung bei postpartal psychisch erkrankten frauen–Evaluation einer interaktionszentrierten Therapie in einer Mutter-Kind-Tagesklinik. PPmP-Psychotherapie· Psychosomatik· Medizinische Psychologie.

[bib0070] Weigl T., Garthus-Niegel S. (2021). [Questionnaires for the Assessment of Peripartum Depression, Anxiety and Stress (Part 1 of a series on psychological assessment during the peripartum period)]. Zeitschrift fur Geburtshilfe und Neonatologie.

[bib0071] Wesselhoeft R., Davidsen K., Sibbersen C., Kyhl H., Talati A., Andersen M.S., Bilenberg N. (2021). Maternal prenatal stress and postnatal depressive symptoms: Discrepancy between mother and teacher reports of toddler psychological problems. Social psychiatry and psychiatric epidemiology.

[bib0072] Williams A.S., Osborn A., Yelland C., Hollamby S. (2021). Changing intergenerational patterns of emotional dysregulation in families with perinatal borderline personality disorder. Archives of Womens Mental Health.

[bib0073] Wittchen H.-U., Zaudig M., Fydrich T. (1997). Skid. Strukturiertes klinisches interview für DSM-IV. Achse I und II. Handanweisung.

[bib0074] Yildiz P.D., Ayers S., Phillips L. (2017). The prevalence of posttraumatic stress disorder in pregnancy and after birth: A systematic review and meta-analysis. Journal of Affective Disorders.

