# Supplementary material for: Treatment of women with postpartum mental disorders in a day clinic mother-baby unit and the effect on child behavioural problems – A 1-year follow-up
Source: Int J Clin Health Psychol. 2025 Jun 11;25(2):100587. doi: 10.1016/j.ijchp.2025.100587 (PMC12197909; doi:10.1016/j.ijchp.2025.100587)
Supplement: Supplementary file 1 [file mmc1.docx]

**Supplementary materials**

**A. Results from dropout analyses**

**Table A.1** Results from dropout analyses for sociodemographic variables using Fisher’s exact tests (*N* = 348).

| Variable | Group | *n (%)* | *p* |
| --- | --- | --- | --- |
| High school diploma | No dropout | 72 (45.0) | .266 |
|  | Dropout | 66 (38.8) |  |
| One child | No dropout | 102 (69.9) | .321 |
|  | Dropout | 94 (63.9) |  |
| Depressive disorders | No dropout | 68 (40.7) | .510 |
|  | Dropout | 67 (37.0) |  |
| Anxiety disorders including OCD and PTSD | No dropout | 47 (28.1) | 1.00 |
|  | Dropout | 50 (27.6) |  |
| Personality disorders | No dropout | 29 (17.4) | .231 |
|  | Dropout | 41 (22.7) |  |
| Schizophrenia/Bipolar disorders | No dropout | 10 (6.0) | .457 |
|  | Dropout | 7 (3.9) |  |
| *Notes*. Two-tailed testing. No dropout participants (*n* = 167) and dropout participants (*n* = 181) both fulfilled inclusion criteria for this study, however dropout participants did not participate at the 1-year follow-up.  OCD = Obsessive compulsive disorder, PTSD = Posttraumatic Stress Disorder | | | |

**Table A.2** Results from dropout analyses for predictors using Welch test (*N* = 348).

| Variable | *t* | *df* | *p* | Difference | 95% CI |
| --- | --- | --- | --- | --- | --- |
| Maternal Age | 0.66 | 344.76 | .512 | .401 | -.80, 1.60 |
| Child Age | -1.82 | 240.63 | .070 | -3.02 | -5.97, 0.44 |
| Gestational week at birth | 0.21 | 213.64 | .836 | 0.06 | -0.51, 0.64 |
| EPDS at admission | -1.46 | 328.82 | .144 | -0.94 | -2.21, 0.32 |
| EPDS at discharge | **-3.19** | **293.31** | **.002** | **-1.77** | **-2.86, -0.68** |
| Change in EPDS admission-discharge | 0.78 | 280.71 | .438 | 0.53 | -0.81, 1.86 |
| STAI at admission | 0.17 | 334.78 | .986 | 0.19 | -2.16, 2.20 |
| STAI at discharge | -1.29 | 303.58 | .197 | -1.47 | -3.71, 0.77 |
| Change in STAI admission-discharge | 1.25 | 293.47 | .212 | 1.39 | -0.79, 3.57 |
| BSI-GSI at admission | -0.82 | 336.97 | .206 | -0.06 | -0.20, 0.08 |
| BSI-GSI at discharge | -1.74 | 300.11 | .083 | -0.10 | -0.21, 0.01 |
| Change in BSI-GSI admission-discharge | 0.40 | 298.00 | .689 | 0.03 | -0.10, 0.15 |
| PSOC at admission | -0.23 | 318.93 | .819 | -0.30 | -2.90, 2.30 |
| PSOC at discharge | 1.02 | 287.61 | .311 | 1.18 | -1.11, 3.48 |
| Change in PSOC admission-discharge | 1.10 | 280.54 | .271 | 1.31 | -1.03, 3.65 |
| PBQ at admission | 0.96 | 329.84 | .337 | 2.27 | -2.37, 6.91 |
| Number of comorbidities | -0.88 | 343.81 | .381 | -0.12 | -0.39, 0.15 |
| Length of hospital stay | **2.44** | **344.59** | **.015** | **0.71** | **0.14, 1.29** |
| *Notes*. Two-tailed testing. No dropout participants (*n* = 167) and dropout participants (*n* = 181) both fulfilled inclusion criteria for this study, however dropout participants did not participate at the 1-year follow-up. EPDS = Edinburgh Postnatal Depression Scale; STAI = State-Trait Anxiety Inventory; BSI-GSI = Brief Symptom Inventory – Global Severity Index; PSOC = Parenting Sense of Competence Scale. PBQ = Parental Bonding Questionnaire. | | | | | |

**B. Descriptive statistics grouped by diagnostic group**

**Table B** Sociodemographic, clinical and birth-related characteristics of mothers according to their primary clinical diagnosis.

|  | Depressive disorders  *n* = 135 | Anxiety disorders (including OCD and PTSD)  *n* = 97 | Personality disorders  *n* = 70 | Schizophrenia or bipolar disorders  *n* = 17 | Test statistics |
| --- | --- | --- | --- | --- | --- |
| *Sociodemographic information* |  |  |  |  |  |
| Age mother in years (mean, SD) | 31.36 (6.02) | 29.31 (5.02) | 28.30 (5.59) | 29.94 (3.31) | F_3,315_ = 5.460, ***p* = <.001** |
| Age child in weeks (mean, SD) | 24.82 (13.26) | 22.37 (12.95) | 23.20 (13.31) | 24.64 (14.72) | F_3,241_ = 0.556, *p* = .65 |
| Gestational week (mean, SD) | 40.10 (1.62) | 39.28 (2.70) | 39.00 (2.39) | 38.69 (3.57) | F_3,206_ = 3.412, ***p* = .018** |
| Preterm birth (n, %) | 5 (5.6) | 7 (10.8) | 8 (19.0) | 2 (15.4) | χ^2^_3_ = 5.95, *p* = .12 |
| Birth mode (n, %) |  |  |  |  |  |
| - Vaginal birth | 79 (80.6) | 52 (78.8) | 42 (82.4) | 13 (92.9) | χ^2^_3_ = 1.57, *p* = .66 |
| - Caesarean section | 19 (19.4) | 14 (21.2) | 9 (17.6) | 1 (7.1) |  |
| Average number of children (mean, SD) | 1.36 (0.62) | 1.40 (0.70) | 1.88 (1.40) | 1.77 (0.73) | F_3,265_ = 5.347, ***p* < .001** |
| Marital status (n, %)   - Married - Single/divorced/widowed | 46 (35.1)  85 (64.9) | 31 (33.3)  62 (66.7) | 12 (18.2)  54 (81.8) | 5 (35.7)  9 (64.3) | χ^2^_3_ = 6.50, *p* = .09 |
| Single mother (n, %) | 29 (22.3) | 15 (16.7) | 27 (43.5) | 1 (7.7) | χ^2^_3_ = 17.51, ***p* < .001** |
| Child custody (n, %)   - Mother - Mother and father | 33 (25.4)  97 (74.6) | 17 (19.3)  71 (80.7) | 31 (48.4)  33 (51.6) | 5 (35.7)  9 (64.3) | χ^2^_3_ = 16.86, ***p* < .001** |
| Education (n, %): High school diploma | 72 (55.0) | 43 (46.2) | 10 (15.2) | 5 (35.7) | χ^2^_3_ = 29.27, ***p* < .001** |
| *Clinical and birth-related information* |  |  |  |  |  |
| Length of MBU stay in weeks (mean, SD) | 8.54 (2.53) | 8.50 (2.66) | 8.68 (3.34) | 10.18 (2.82) | F_3,315_ = 1.87, *p* = .13 |
| Number of mental comorbidities (mean, SD) | 1.43 (1.21) | 1.85 (1.04) | 2.54 (1.24) | 1.47 (1.50) | F_3,315_ = 14.02, ***p* < .001** |
| Somatic diseases (self-report, n, %) | 89 (66.9) | 66 (69.5) | 52 (77.6) | 13 (81.3) | χ^2^_3_ = 3.40, *p* = .34 |
| Medication intake during day clinic stay (n, %)   - Psychotropic medication - Other medication | 117 (88.0)  93 (70.5)  78 (59.1) | 72 (77.4)  42 (45.7)  46 (50.0) | 45 (68.2)  30 (45.5)  33 (50.0) | 16 (100)  16 (100)  5 (31.3) | χ^2^_3_ = 15.88, ***p* < .001**  χ^2^_3_ = 30.09, ***p* < .001**  χ^2^_3_ = 5.57, *p* = .14 |
| Parent-child bonding (PBQ) |  |  |  |  |  |
| - At admission (mean, SD) | 38.21 (23.03) | 27.27 (16.72) | 27.57 (20.45) | 23.44 (21.37) | F_3,303_ = 7.50, ***p* = <.001** |
| - At discharge (mean, SD) | 15.86 (10.15) | 13.90 (8.63) | 16.27 (11.37) | 14.27 (12.33) | F_3,274_ = 0.90, *p* = .44 |
| *1-year follow-up information* |  |  |  |  |  |
| Child behaviour problems (CBCL) at 1-year follow-up (mean, SD) |  |  |  |  |  |
| - Total score (mean, SD) | 27.12 (15.04) | 23.22 (14.90) | 24.55 (13.86) | 24.10 (10.99) | F_3,147_ = 0.70, *p* = .55 |
| - Externalizing symptoms (mean, SD) | 12.21 (6.91) | 9.69 (6.20) | 9.31 (5.24) | 10.70 (5.01) | F_3,147_ = 2.12, *p* = .10 |
| - Internalizing symptoms (mean, SD) | 5.30 (3.53) | 5.20 (4.15) | 6.15 (4.96) | 4.60 (3.66) | F_3,144_ = 0.49, *p* = .69 |
| Continued treatment or received assistance after discharge (self-report at 1-year follow-up)^a^ (n, %) | 65 (95.6) | 46 (97.9) | 29 (100) | 10 (100) | χ^2^_3_ = 1.97, *p* = .66 |

*Notes*. *N* varied slightly due to missing values.

OCD = Obsessive compulsive disorder, PTSD = Posttraumatic Stress Disorder, PBQ = Parental Bonding Questionnaire, CBCL = Child’s Behaviour Checklist, MBU = mother baby unit

^a^ outpatient psychotherapy at the University Hospital / with a registered therapist, medication treatment at the University Hospital / with a registered doctor, inpatient treatment, day clinic treatment, outpatient mother-child bonding work (e.g., occupational therapy), extended midwife assistance, domestic help, or support from the youth welfare office.

**C. Diagnostic group at admission, discharge and follow-up**

**Table C** Estimated marginal means at admission, discharge, and 1-year follow-up and averaged across all time points according to diagnostic group (*N* = 319).

|  | **Admission** | **Discharge** | **1-year Follow-up** | **Averaged across time** |
| --- | --- | --- | --- | --- |
| **EPDS** | *M (SE)* | *M (SE)* | *M (SE)* | *M (SE)* |
| Dep | 16.19 (0.47) | 8.77 (0.48) | 9.15 (0.61) | 11.33 (0.38) |
| Anx | 15.61 (0.55) | 9.85 (0.56) | 7.87 (0.73) | 11.27 (0.44) |
| Sch/Bip | 13.22 (1.29) | 9.16 (1.36) | 7.76 (1.61) | 9.95 (1.04) |
| Pers | 17.42 (0.65) ^a^ | 11.65 (0.70) ^b^ | 11.52 (0.93) ^c^ | 13.38 (0.53) ^d^ |
| ^a^ *Pers > Schiz/Bip (*FDR-corrected *p = .023),* ^b^ Pers > Dep (FDR-corrected *p* = .014), *^c^ Pers > Anx (*FDR-corrected *p = .019),* ^d^ Pers > Dep, Anx, *Schiz/Bip* (FDR-corrected *p’s* < .01) | | | | |
| **STAI-T** | *M (SE)* | *M (SE)* | *M (SE)* | *M (SE)* |
| Dep | 55.2 (0.88) | 45.7 (0.91) | 45.5 (1.14) ^f^ | 48.6 (0.74) |
| Anx | 55.1 (1.03) | 46.4 (1.07) | 41.8 (1.38) | 48.2 (0.88) |
| Sch/Bip | 50.0 (2.45) | 45.2 (2.57) | 36.4 (2.99) | 44.3 (2.06) |
| Pers | 57.4 (1.22) ^e^ | 48.2 (1.32) | 49.7 (1.71) ^g^ | 51.3 (1.05) ^h^ |
| *^e^ Pers > Schiz/Bip (*FDR-corrected p *= .032),* *^f^ Dep > Sch/Bip (*FDR-corrected p *= .026),* ^g^ Pers > Sch/Bip, *Anx* (FDR-corrected *p* < .003), ^h^ Pers > Sch/Bip (FDR-corrected *p* = .017) | | | | |
| **BSI-GSI** | *M (SE)* | *M (SE)* | *M (SE)* | *M (SE)* |
| Dep | 1.21 (0.05) | 0.60 (0.05) | 0.66 (0.06) | 0.81 (0.04) |
| Anx | 1.32 (0.06) | 0.72 (0.06) | 0.59 (0.08) | 0.89 (0.05) |
| Sch/Bip | 0.84 (0.14) ^i^ | 0.73 (0.15) | 0.37 (0.17) | 0.65 (0.12) |
| Pers | 1.52 (0.07) ^j^ | 0.94 (0.07) ^k^ | 0.95 (0.10) ^l^ | 1.13 (0.06) ^m^ |
| ^i^ Schiz/Bip < *Anx*, Dep, *Pers* (FDR-corrected *p <* .028), *^j^ Pers > Anx, Dep (*FDR-corrected *p < .048),* *^k^ Pers > Anx, Dep* (FDR-corrected *p’s < .046),* ^l^ Pers > Anx, *Dep, Schiz/Bip* (FDR-corrected *p* < .028), ^m^ Pers > Dep, Anx, Sch/Bip (FDR-corrected *p’s* < .01) | | | | |
| **PSOC** | *M (SE)* | *M (SE)* | *M (SE)* | *M (SE)* |
| Dep | 56.2 (0.94) ^n^ | 66.6 (0.96) | 66.2 (1.19) | 62.9 (0.80) ^p^ |
| Anx | 59.8 (1.11) | 68.5 (1.14) | 71.2 (1.42) | 66.2 (0.95) |
| Sch/Bip | 65.6 (2.67) | 70.7 (2.79) | 71.1 (3.13) | 69.4 (2.28) |
| Pers | 58.9 (1.33) | 67.2 (1.40) | 64.1 (1.77) ^o^ | 64.0 (1.14) |
| ^n^ Dep < Sch/Bip (FDR-corrected *p* = .016), ^o^ Pers < Anx (FDR-corrected *p =* .018), ^p^ Dep < Anx, *Schiz/Bip* (FDR-corrected *p <* .030) | | | | |
| *Note*. FDR-corrected *p* values employed per 18 comparisons across three time points (admission, discharge, 1-year follow-up) per measure and per 6 comparisons averaged across time. Results in italics indicate diverging results using MI data, see Table D5 for comparison. Dep = Depressive disorders. Anx = Anxiety disorders/OCD/PTSD. Pers = Personality disorders. Sch/Bip = Schizophrenia/Bipolar disorders. EPDS = Edinburgh Postnatal Depression Scale. STAI-*T* = State-Trait Anxiety Inventory – Trait Subscale. BSI-GSI = Brief Symptom Inventory - Global Severity Index. PSOC = Parenting Sense of Competence Scale. | | | | |

**D. Sensitivity analysis with multiple imputed data**

To handle missing data, we used multiple imputation by chained equations (MICE) as implemented in the *mice* package in R (van Buuren & Groothuis-Oudshoorn, 2011). We generated 100 imputed datasets using 100 iterations per imputation cycle to ensure convergence and stable parameter estimates to impute missing values (Wijesuriya et al., 2025; see supplements S1 for exact percentages). Predictive Mean Matching (pmm) was used as the imputation method to preserve the distribution of observed values and avoid unrealistic imputations. The imputation model contained diagnostic group, treatment duration, clinical outcomes at all three time points, maternal education, maternal age, child age in weeks, gestational week at birth, number of children, number of comorbidities, preterm birth, mother-child bonding at admission and discharge, single parent, relationship status, CBCL score (total, externalising, internalising), assuming the data were missing at random. The imputed datasets were then analysed according to Rubin’s rules (Rubin, 2004).

**Table D1** Raw symptom scores for depressive (EPDS), anxiety (STAI), and general symptoms (BSI-GSI), and parental sense of competence (PSOC) at admission, discharge, and 1-year follow-up among mothers with postpartum mental disorders (*N* = 348) and results from post-hoc comparisons between assessment points with FDR-corrected *p*-values.

|  | **Admission (T0)** | | **Discharge (T1)** | | **1-year follow-up (T2)** | |  |  | **Pairwise comparisons^a^** | | | | |
| --- | --- | --- | --- | --- | --- | --- | --- | --- | --- | --- | --- | --- | --- |
|  | *M* (*SD*) | % above cut-off | *M* (*SD*) | % above cut-off | *M* (*SD*) | % above cut-off | ICC |  |  | *b* | *SE(b)* | *t* | *P_FDR_* |
| **EPDS** | 15.82 (5.9) | 83.7 | 9.76 (4.9) | 50.0 | 8.92 (5.4) | 44.3 | .17 |  | T0 – T1 | **6.06** | **0.34** | **17.58** | **< .001** |
|  | *(n =* 331) |  | *(n =* 306) |  | *(n =* 167) |  |  |  | T0 – T2 | **6.29** | **0.42** | **15.10** | **< .001** |
|  |  |  |  |  |  |  |  |  | T1 – T2 | 0.23 | 0.42 | 0.56 | .576 |
|  |  |  |  |  |  |  |  |  |  |  |  |  |  |
| **STAI** | 54.77 (10.2) | 79.3 | 46.68 (10.0) | 50.0 | 44.56 (11.8) | 42.7 | .34 |  | T0 – T1 | **8.32** | **0.62** | **13.42** | **< .001** |
|  | *(n =* 338) |  | *(n =* 306) |  | *(n =* 164) |  |  |  | T0 – T2 | **9.25** | **0.78** | **11.81** | **< .001** |
|  |  |  |  |  |  |  |  |  | T1 – T2 | 0.93 | 0.79 | 1.17 | .245 |
|  |  |  |  |  |  |  |  |  |  |  |  |  |  |
| **BSI-GSI** | 1.26 (0.67) | 82.6 | 0.71 (0.49) | 50.5 | 0.66 (0.55) | 41.3 | .32 |  | T0 – T1 | **0.55** | **0.03** | **17.05** | **< .001** |
|  | *(n =* 339) |  | *(n =* 306) |  | *(n =* 167) |  |  |  | T0 – T2 | **0.57** | **0.04** | **14.72** | **< .001** |
|  |  |  |  |  |  |  |  |  | T1 – T2 | 0.02 | 0.04 | 0.49 | .623 |
|  |  |  |  |  |  |  |  |  |  |  |  |  |  |
| **PSOC** | 59.03 (11.9) | N/A | 67.28 (10.0) | N/A | 67.72 (10.4) | N/A | .36 |  | T0 – T1 | **-8.49** | **0.64** | **-13.33** | **< .001** |
|  | *(n =* 322) |  | *(n =* 298) |  | *(n =* 164) |  |  |  | T0 – T2 | **-8.74** | **0.78** | **-11.23** | **< .001** |
|  |  |  |  |  |  |  |  |  | T1 – T2 | -0.25 | 0.78 | -0.32 | .753 |
|  |  |  |  |  |  |  |  |  |  |  |  |  |  |
| *Note*. A positive *b* means that at Time 0 the score was higher than at Time 1 (e.g., T0 – T1). A negative *b* means that at Time 0 the score was lower than at Time 1 (e.g., T0 – T1). EPDS = Edinburgh Postnatal Depression Scale. STAI = State-Trait Anxiety Inventory – Trait Subscale. BSI-GSI = Brief Symptom Inventory – Global Severity Index. PSOC = Parenting Sense of Competence Scale. ICC = Intraclass Correlation Coefficient from an empty means linear mixed model. T0 = Admission. T1 = Discharge. T2 = 1-year follow-up. ^a^ Results are based on 100 imputed datasets using multiple imputation. Estimates and standard errors were pooled using Rubin’s rules. Results based on linear mixed model with time and treatment duration as a fixed effect. | | | | | | | | | | | | | |

**Table D2** Results from post-hoc comparisons regarding clinical outcomes averaged across admission, discharge and 1-year follow-up between diagnostic groups with FDR-corrected p-values (N = 319) based on the multiple imputed data.

|  | *b* | *SE (b)* | *t* | *P_FDR_* |
| --- | --- | --- | --- | --- |
| **EPDS** |  |  |  |  |
| Dep – Anx | 0.05 | 0.58 | 0.08 | .933 |
| Dep – Pers | **-2.17** | **0.64** | **-3.39** | **.004** |
| Dep – Sch/Bip | 1.14 | 1.07 | 1.06 | .393 |
| Anx – Pers | **-2.21** | **0.69** | **-3.20** | **.004** |
| Anx – Sch/Bip | 1.09 | 1.11 | 0.98 | .393 |
| Pers – Schiz/Bip | **3.31** | **1.13** | **2.92** | **.007** |
|  |  |  |  |  |
| **STAI-T** |  |  |  |  |
| Dep – Anx | 0.65 | 1.20 | 0.54 | .587 |
| Dep – Pers | -2.90 | 1.33 | -2.17 | .061 |
| Dep – Sch/Bip | 4.54 | 2.22 | 2.05 | .061 |
| Anx – Pers | **-3.55** | **1.45** | **-2.45** | **.045** |
| Anx – Sch/Bip | 3.89 | 2.30 | 1.69 | .109 |
| Pers – Schiz/Bip | **7.43** | **2.36** | **3.15** | **.010** |
|  |  |  |  |  |
| **BSI-GSI** |  |  |  |  |
| Dep – Anx | -0.06 | 0.06 | -1.04 | .298 |
| Dep – Pers | **-0.31** | **0.07** | **-4.35** | **< .001** |
| Dep – Sch/Bip | 0.15 | 0.12 | 1.27 | .244 |
| Anx – Pers | **-0.22** | **0.08** | **-3.24** | **.002** |
| Anx – Sch/Bip | 0.22 | 0.12 | 1.78 | .113 |
| Pers – Schiz/Bip | **0.46** | **0.13** | **3.68** | **<.001** |
|  |  |  |  |  |
| **PSOC** |  |  |  |  |
| Dep – Anx | **-3.35** | **1.22** | **-2.84** | **.028** |
| Dep – Pers | -0.46 | 1.34 | -0.34 | .734 |
| Dep – Sch/Bip | -4.88 | 2.44 | -2.00 | .069 |
| Anx – Pers | 3.00 | 1.44 | 2.08 | .069 |
| Anx – Sch/Bip | -1.88 | 2.35 | -0.80 | .508 |
| Pers – Schiz/Bip | -4.88 | 2.44 | -2.00 | .069 |
|  |  |  |  |  |
| *Note*. Results are based on 100 imputed datasets using multiple imputation. Estimates and standard errors were pooled using Rubin’s rules. A positive *b* means that Group 1 had a higher score than Group 2 (Group 1 – Group 2). A negative *b* means that Group 1 had a lower score than Group 2 (Group 1 – Group 2). Dep = Depressive disorders. Anx = Anxiety disorders/OCD/PTSD. Pers = Personality disorders. Sch/Bip = Schizophrenia/Bipolar disorders. EPDS = Edinburgh Postnatal Depression Scale. STAI-T = State-Trait Anxiety Inventory – Trait Subscale. BSI-GSI = Brief Symptom Inventory – Global Severity Index. PSOC = Parenting Sense of Competence Scale. | | | | |

**Table D3** Results from post-hoc comparisons of change from admission to discharge and discharge to 1-year follow-up between diagnostic groups with FDR-corrected p-values (N = 330).

|  | **Admission to Discharge** | | | |  | **Discharge to 1-year follow-up** | | | |
| --- | --- | --- | --- | --- | --- | --- | --- | --- | --- |
|  | *b* | *SE (b)* | *t* | *P_FDR_* |  | *b* | *SE (b)* | *t* | *P_FDR_* |
| **EPDS** |  |  |  |  |  |  |  |  |  |
| Dep – Anx | -1.50 | 0.83 | -1.80 | .151 |  | 1.50 | 1.07 | 1.40 | .902 |
| Dep – Pers | -1.70 | 0.96 | -1.78 | .151 |  | 0.28 | 1.21 | 0.23 | .917 |
| Dep – Sch/Bip | -3.18 | 1.61 | -1.98 | .151 |  | 1.31 | 1.81 | 0.72 | .902 |
| Anx – Pers | -0.20 | 1.00 | -0.20 | .843 |  | -1.22 | 1.28 | -0.95 | .902 |
| Anx – Sch/Bip | -1.67 | 1.64 | -1.02 | .461 |  | -0.20 | 1.88 | -0.10 | .917 |
| Pers – Schiz/Bip | -1.47 | 1.70 | -0.87 | .463 |  | 1.02 | 1.95 | 0.52 | .902 |
| **STAI-T** |  |  |  |  |  |  |  |  |  |
| Dep – Anx | -0.57 | 1.49 | -0.39 | .915 |  | 2.94 | 2.07 | 1.42 | .189 |
| Dep – Pers | -0.38 | 1.68 | -0.23 | .915 |  | -1.00 | 2.28 | -0.44 | .664 |
| Dep – Sch/Bip | -4.39 | 2.85 | -1.54 | .377 |  | **8.05** | **3.33** | **2.42** | **.048** |
| Anx – Pers | 0.19 | 1.78 | 0.11 | .915 |  | -3.93 | 2.43 | -1.62 | .189 |
| Anx – Sch/Bip | -3.81 | 2.90 | -1.32 | .377 |  | 5.11 | 3.55 | 1.44 | .189 |
| Pers – Schiz/Bip | -4.00 | 3.01 | -1.33 | .377 |  | **9.04** | **3.65** | **2.48** | **.048** |
| **BSI-GSI** |  |  |  |  |  |  |  |  |  |
| Dep – Anx | -0.01 | 0.08 | -0.14 | .991 |  | 0.14 | 0.10 | 1.45 | .294 |
| Dep – Pers | -0.01 | 0.09 | -0.13 | .991 |  | 0.02 | 0.11 | 0.19 | .849 |
| Dep – Sch/Bip | **-0.47** | **0.15** | **-3.13** | **.008** |  | 0.35 | 0.17 | 2.07 | .216 |
| Anx – Pers | -0.00 | 0.10 | -0.01 | .991 |  | -0.12 | 0.12 | -1.02 | .373 |
| Anx – Sch/Bip | **-0.46** | **0.15** | **-2.99** | **.008** |  | 0.21 | 0.18 | 1.20 | .346 |
| Pers – Schiz/Bip | **-0.46** | **0.16** | **-2.89** | **.008** |  | 0.33 | 0.18 | 1.80 | .216 |
| **PSOC** |  |  |  |  |  |  |  |  |  |
| Dep – Anx | 1.61 | 1.50 | 1.07 | .427 |  | -2.55 | 1.94 | -1.31 | .381 |
| Dep – Pers | 1.51 | 1.73 | 0.87 | .459 |  | 3.00 | 2.11 | 1.43 | .381 |
| Dep – Sch/Bip | 5.81 | 2.91 | 2.00 | .277 |  | -0.30 | 3.38 | -0.09 | .929 |
| Anx – Pers | -0.09 | 1.81 | -0.05 | .959 |  | 5.55 | 2.33 | 2.39 | .107 |
| Anx – Sch/Bip | 4.20 | 2.98 | 1.41 | .329 |  | 2.25 | 3.48 | 0.65 | .623 |
| Pers – Schiz/Bip | 4.30 | 3.09 | 1.39 | .329 |  | -3.30 | 3.79 | -0.89 | .559 |
| *Note*. Results are based on 100 imputed datasets using multiple imputation. Estimates and standard errors were pooled using Rubin’s rules. A negative *b* means that Group 1 had a greater reduction than Group 2 for the respective time point (Group 1 – Group 2). A positive *b* means that Group 2 had a greater reduction than Group 1 (Group 1 – Group 2). Dep = Depressive disorders. Anx = Anxiety disorders/OCD/PTSD. Pers = Personality disorders. Sch/Bip = Schizophrenia/Bipolar disorders. EPDS = Edinburgh Postnatal Depression Scale. STAI-T = State-Trait Anxiety Inventory – Trait Subscale. BSI-GSI = Brief Symptom Inventory – Global Severity Index. PSOC = Parenting Sense of Competence Scale. | | | | | | | | | |

**Table D4** Results of multiple regression analyses predicting overall child behaviour problems (CBCL) from maternal symptom change across treatment and until 1-year follow-up.

|  | $\beta$ | *B* | *SE* | *p* | *R^2^* |
| --- | --- | --- | --- | --- | --- |
| **Depressive symptoms (EPDS)** |  |  |  |  |  |
| **Admission to discharge**^1^ |  |  |  |  |  |
| *Model 1 ^a^:* |  |  |  |  | .03 |
| Admission to discharge EPDS | -.12 | -0.28 | 0.25 | .258 |  |
| *Model 2 ^a^:* |  |  |  |  | .09 |
| Admission to discharge EPDS | -.01 | -0.03 | 0.26 | .901 |  |
| 1-year follow-up EPDS | **.27** | **0.69** | **0.22** | **.002** |  |
|  |  |  |  |  |  |
| **Admission to 1-year follow-up** |  |  |  |  |  |
| *Model 1 ^a^:* |  |  |  |  | .09 |
| Admission to 1-year follow-up EPDS | **-.34** | **-0.70** | **0.20** | **< .001** |  |
|  |  |  |  |  |  |
| **Anxiety symptoms (STAI-T)** |  |  |  |  |  |
| **Admission to discharge** ^2^ |  |  |  |  |  |
| *Model 1 ^a^:* |  |  |  |  | .03 |
| Admission to discharge STAI-T | -.09 | -0.13 | 0.12 | .297 |  |
| *Model 2 ^a^:* |  |  |  |  | .12 |
| Admission to discharge STAI-T | .02 | 0.03 | 0.13 | .812 |  |
| 1-year follow-up STAI-T | **.33** | **0.41** | **0.11** | **<.001** |  |
|  |  |  |  |  |  |
| **Admission to 1-year follow-up** |  |  |  |  |  |
| *Model 1 ^a^:* |  |  |  |  | .11 |
| Admission to 1-year follow-up STAI-T | **-.34** | **-0.40** | **0.10** | **< .001** |  |
|  |  |  |  |  |  |
| **General psychological distress (BSI-GSI)** |  |  |  |  |  |
| **Admission to discharge** ^3^ |  |  |  |  |  |
| *Model 1 ^a^:* |  |  |  |  | .04 |
| Admission to discharge BSI-GSI | -.18 | -4.53 | 2.53 | .076 |  |
| *Model 2 ^a^:* |  |  |  |  | .13 |
| Admission to discharge BSI-GSI | .02 | 0.62 | 2.90 | .831 |  |
| 1-year follow-up BSI-GSI | **.37** | **9.46** | **2.45** | **<.001** |  |
|  |  |  |  |  |  |
| **Admission to 1-year follow-up** |  |  |  |  |  |
| *Model 1 ^a^:* |  |  |  |  | .12 |
| Admission to 1-year follow-up BSI-GSI | **-.44** | **-9.23** | **2.04** | **< .001** |  |
|  |  |  |  |  |  |
| **Parental sense of competence (PSOC)** |  |  |  |  |  |
| **Admission to discharge**^4^ |  |  |  |  |  |
| *Model 1 ^a^:* |  |  |  |  | .06 |
| Admission to discharge PSOC | -.08 | -0.11 | 0.13 | .363 |  |
| *Model 2 ^a^:* |  |  |  |  | .12 |
| Admission to discharge PSOC | .02 | 0.03 | 0.13 | .834 |  |
| 1-year follow-up PSOC | **-.28** | **-0.38** | **0.12** | **.002** |  |
|  |  |  |  |  |  |
| **Admission to 1-year follow-up** |  |  |  |  |  |
| *Model 1 ^a^:* |  |  |  |  | .12 |
| Admission to 1-year follow-up PSOC | **-.32** | **-0.36** | **0.11** | **< .001** |  |
| *Note*. Results are based on 100 imputed datasets using multiple imputation. Estimates and standard errors were pooled using Rubin’s rules. Change scores from admission to discharge and admission to 1-year follow-up were calculated such that a negative regression coefficient signifies that a greater improvement in maternal symptoms was associated with lower CBCL scores. CBCL = Child’s Behaviour Checklist, EPDS = Edinburgh Postnatal Depression Scale. STAI-*T* = State-Trait Anxiety Inventory – Trait Subscale. BSI-GSI = Brief Symptom Inventory – Global Severity Index. PSOC = Parenting Sense of Competence Scale. | | | | | |

**Table D5** Estimated marginal means at admission, discharge, and 1-year follow-up and averaged across all time points according to diagnostic group (*N* = 319).

|  | **Admission** | **Discharge** | **1-year follow-up** | **Averaged across time** |
| --- | --- | --- | --- | --- |
| **EPDS** | *M (SE)* | *M (SE)* | *M (SE)* | *M (SE)* |
| Dep | 16.12 (0.46) | 8.82 (0.47) | 9.05 (0.62) | 11.33 (0.37) |
| Anx | 15.58 (0.55) | 9.77 (0.55) | 8.50 (0.75) | 11.28 (0.44) |
| Sch/Bip | 13.31 (1.29) | 9.18 (1.33) | 8.10 (1.67) | 10.20 (1.01) |
| Pers | 17.26 (0.64) | 11.65 (0.69) ^a^ | 11.60 (0.91) | 13.50 (0.53) ^b^ |
| ^a^ Pers > Dep (FDR-corrected *p* < .001), ^b^ Pers > Dep, Anx (FDR-corrected *p’s* < .01) | | | | |
| **STAI-T** | *M (SE)* | *M (SE)* | *M (SE)* | *M (SE)* |
| Dep | 54.96 (0.89) | 45.70 (0.92) | 45.72 (1.20) | 48.79 (0.76) |
| Anx | 54.90 (1.05) | 46.22 (1.07) | 43.31 (1.53) | 48.14 (0.92) |
| Sch/Bip | 50.17 (2.51) | 45.31 (2.59) | 37.28 (2.97) | 44.25 (2.09) |
| Pers | 57.26 (1.23) | 48.39 (1.33) | 49.41 (1.89) ^c^ | 51.69 (1.12) ^d^ |
| ^c^ Pers > Sch/Bip (FDR-corrected *p* = .012), ^d^ Pers > Anx, Sch/Bip (FDR-corrected *p’s* < .04) | | | | |
| **BSI-GSI** | *M (SE)* | *M (SE)* | *M (SE)* | *M (SE)* |
| Dep | 1.21 (0.05) | 0.60 (0.05) | 0.64 (0.06) | 0.81 (0.04) |
| Anx | 1.31 (0.06) | 0.72 (0.06) | 0.62 (0.07) | 0.88 (0.05) |
| Sch/Bip | 0.86 (0.14) ^e^ | 0.73 (0.14) | 0.42 (0.15) | 0.67 (0.11) |
| Pers | 1.52 (0.07) ^f^ | 0.93 (0.07) ^g^ | 0.94 (0.09) ^h^ | 1.13 (0.06) ^i^ |
| ^e^ Schiz/Bip < Anx, Dep (FDR-corrected *p <* .034), ^f^ Pers > Anx, Dep, Schiz/Bip (FDR-corrected *p* < .040), ^g^ Pers > Anx, Dep (FDR-corrected *p’s* < .045), ^h^ Pers > Anx, Dep, Schiz/Bip (FDR-corrected *p* < .020), ^i^ Pers > Dep, Anx, Sch/Bip (FDR-corrected *p’s* < .01) | | | | |
| **PSOC** | *M (SE)* | *M (SE)* | *M (SE)* | *M (SE)* |
| Dep | 56.34 (0.92) ^j^ | 66.54 (0.93) | 66.84 (1.12) | 63.24 (0.77) ^l^ |
| Anx | 60.02 (1.09) | 68.61 (1.11) | 71.46 (1.50) | 66.70 (0.94) |
| Sch/Bip | 65.46 (2.60) | 69.84 (2.71) | 70.44 (3.01) | 68.58 (2.17) |
| Pers | 58.81 (1.30) | 67.49 (1.36) | 64.79 (1.74) ^k^ | 63.70 (1.11) |
| ^j^ Dep < Sch/Bip (FDR-corrected *p* = .017), ^k^ Pers < Anx (FDR-corrected *p =* .031), ^l^ Dep < Anx (FDR-corrected *p =* .028) | | | | |
| *Note*. FDR-corrected *p* values employed per 18 comparisons across three time point per measure. Dep = Depressive disorders. Anx = Anxiety disorders/OCD/PTSD. Pers = Personality disorders. Sch/Bip = Schizophrenia/Bipolar disorders. EPDS = Edinburgh Postnatal Depression Scale. STAI-T = State-Trait Anxiety Inventory – Trait Subscale. BSI-GSI = Brief Symptom Inventory – Global Severity Index. PSOC = Parenting Sense of Competence Scale. | | | | |

**Figure D1** Line graph showing estimated mean clinical outcome scores at admission, discharge, and 1-year follow-up grouped by diagnostic group with standard error bars based on 100 imputed datasets using multiple imputation.


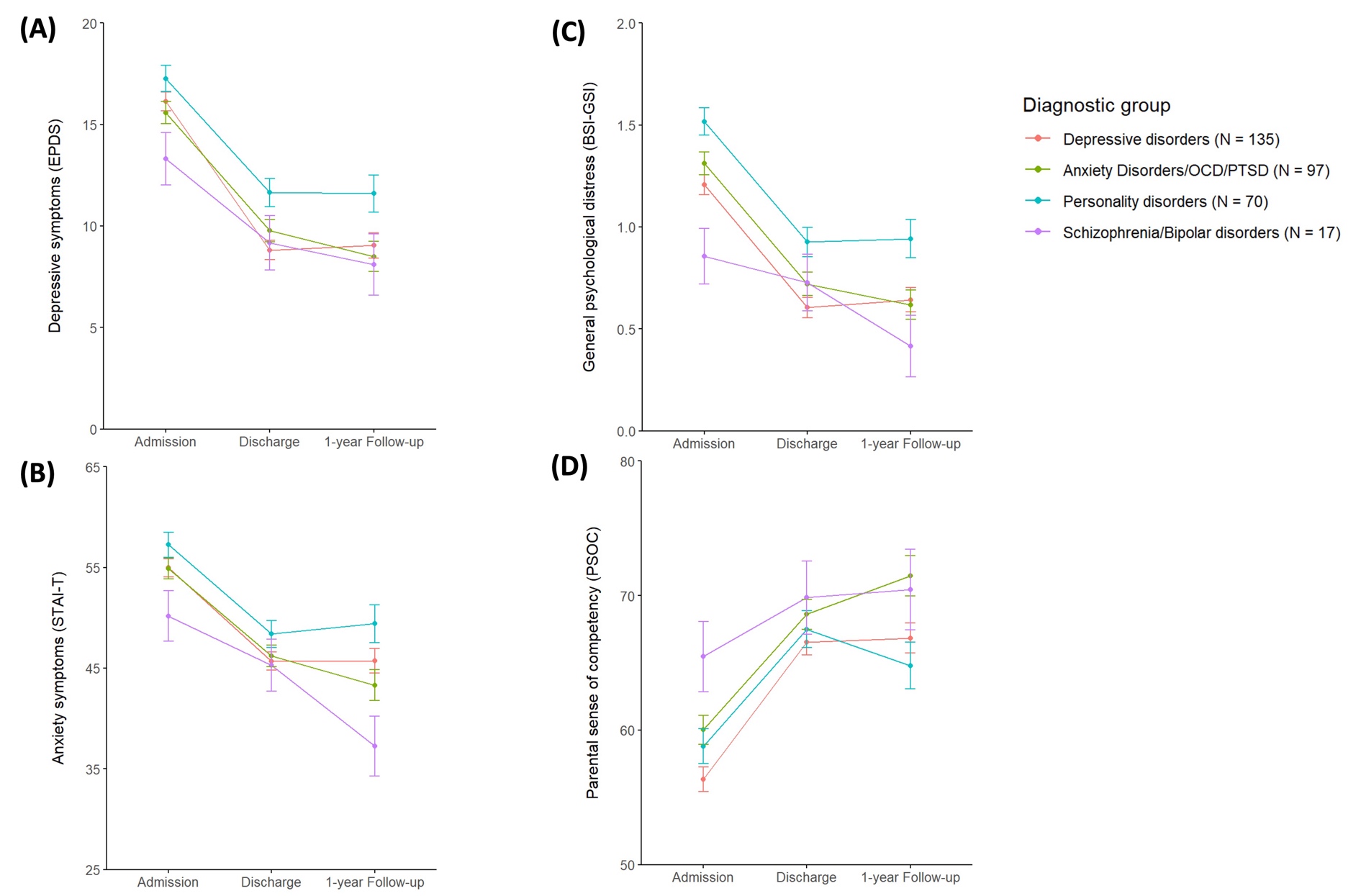


**E: Calculation of the reliable change index for the present sample** (according to the method described by Jacobson & Truax, 1992 and Matthey, 2004)

1. **EPDS**

*Cohen’s alpha for EPDS = 0.87 (Cox et al., 1987)*

*average SD from data at admission = 5.9*

$${RCI=\frac{{(X}_{2}-X_{1})}{S_{diff}} where S}_{diff}=\sqrt{\left( 2(S_{EM})^2 \right)}$$

$$S_{EM}=S_{1}\sqrt{\left( 1-r_{xx} \right)}$$

$$S_{EM}=5.9\sqrt{\left( 1-0.87 \right)}$$

$$S_{EM}=2.13$$

**Thus, the standard error of difference is:**

$$S_{diff}= \sqrt{\left( 2(S_{EM})^2 \right)}$$

$$S_{diff}= \sqrt{\left( 2(2.13)^2 \right)}$$

$$S_{diff}= 3.01$$

**Thus, to get an RCI of 1.96 or more,** $\boldsymbol{(X}_{\boldsymbol{2}}\boldsymbol{-}\boldsymbol{X}_{\boldsymbol{1}}\boldsymbol{)}$ **has to be at least:**

$$RCI=\frac{{(X}_{2}-X_{1})}{S_{diff}}$$

$$RCI=\frac{{(X}_{2}-X_{1})}{3.01}$$

$$1.96 x 3.01=(X_{2}- X_{1})$$

$$1.96 x 3.01=5.90$$

1. **STAI-T**

*Cohen’s alpha for STAI-T = .94 (Grant et al., 2008)*

*average SD from data at admission = 10.2*

$${RCI=\frac{{(X}_{2}-X_{1})}{S_{diff}} where S}_{diff}=\sqrt{\left( 2(S_{EM})^2 \right)}$$

$$S_{EM}=S_{1}\sqrt{\left( 1-r_{xx} \right)}$$

$$S_{EM}=10.2\sqrt{\left( 1-0.94 \right)}$$

$$S_{EM}=2.50$$

Thus, the standard error of difference is:

$$S_{diff}= \sqrt{\left( 2(S_{EM})^2 \right)}$$

$$S_{diff}= \sqrt{\left( 2(2.50)^2 \right)}$$

$$S_{diff}= 3.53$$

Thus, to get an RCI of 1.96 or more, ${(X}_{2}-X_{1})$ has to be at least:

$$RCI=\frac{{(X}_{2}-X_{1})}{S_{diff}}$$

$$RCI=\frac{{(X}_{2}-X_{1})}{3.53}$$

$$1.96 x 3.53=(X_{2}- X_{1})$$

$$1.96 x 3.53=6.92$$

1. **BSI-GSI**

*Cohen’s alpha for BSI-GSI = .96 (Prinz et al., 2013)*

*average SD from data across admission, discharge, follow-up = 0.67*

$${RCI=\frac{{(X}_{2}-X_{1})}{S_{diff}} where S}_{diff}=\sqrt{\left( 2(S_{EM})^2 \right)}$$

$$S_{EM}=S_{1}\sqrt{\left( 1-r_{xx} \right)}$$

$$S_{EM}=0.67\sqrt{\left( 1-0.96 \right)}$$

$$S_{EM}=0.134$$

Thus, the standard error of difference is:

$$S_{diff}= \sqrt{\left( 2(S_{EM})^2 \right)}$$

$$S_{diff}= \sqrt{\left( 2(0.134)^2 \right)}$$

$$S_{diff}= 0.19$$

Thus, to get an RCI of 1.96 or more, ${(X}_{2}-X_{1})$ has to be at least:

$$RCI=\frac{{(X}_{2}-X_{1})}{S_{diff}}$$

$$RCI=\frac{{(X}_{2}-X_{1})}{0.19}$$

$$1.96 x 0.19=(X_{2}- X_{1})$$

$$1.96 x 0.19=0.37$$

1. **PSOC**

*Cohen’s alpha for PSOC = .93 (Rahimzadeh et al., 2024)*

*average SD from data across admission, discharge, follow-up = 11.9*

$${RCI=\frac{{(X}_{2}-X_{1})}{S_{diff}} where S}_{diff}=\sqrt{\left( 2(S_{EM})^2 \right)}$$

$$S_{EM}=S_{1}\sqrt{\left( 1-r_{xx} \right)}$$

$$S_{EM}=11.9\sqrt{\left( 1-0.93 \right)}$$

$$S_{EM}=3.15$$

Thus, the standard error of difference is:

$$S_{diff}= \sqrt{\left( 2(S_{EM})^2 \right)}$$

$$S_{diff}= \sqrt{\left( 2(3.15)^2 \right)}$$

$$S_{diff}= 4.45$$

Thus, to get an RCI of 1.96 or more, ${(X}_{2}-X_{1})$ has to be at least:

$$RCI=\frac{{(X}_{2}-X_{1})}{S_{diff}}$$

$$RCI=\frac{{(X}_{2}-X_{1})}{4.45}$$

$$1.96 x 4.45=(X_{2}- X_{1})$$

$$1.96 x 4.45=8.73$$

**References**

Cox, J. L., Holden, J. M., & Sagovsky, R. (1987). Detection of postnatal depression. Development of the 10-item Edinburgh Postnatal Depression Scale. *The British Journal of Psychiatry: The Journal of Mental Science*, *150*, 782–786. https://doi.org/10.1192/bjp.150.6.782

Grant, K.-A., McMahon, C., & Austin, M.-P. (2008). Maternal anxiety during the transition to parenthood: A prospective study. *Journal of Affective Disorders*, *108*(1–2), 101–111. https://doi.org/10.1016/j.jad.2007.10.002

Jacobson, N. S., & Truax, P. (1992). Clinical significance: A statistical approach to defining meaningful change in psychotherapy research. In A. E. Kazdin (Ed.), *Methodological issues & strategies in clinical research.* (pp. 631–648). American Psychological Association. https://doi.org/10.1037/10109-042

Matthey, S. (2004). Calculating clinically significant change in postnatal depression studies using the Edinburgh Postnatal Depression Scale. Journal of Affective Disorders, 78(3), 269-272. https://doi.org/10.1016/S0165-0327(02)00313-0

Prinz, U., Nutzinger, D. O., Schulz, H., Petermann, F., Braukhaus, C., & Andreas, S. (2013). Comparative psychometric analyses of the SCL-90-R and its short versions in patients with affective disorders. *BMC Psychiatry*, *13*(1), 104. https://doi.org/10.1186/1471-244X-13-104

Rahimzadeh, M., Saeieh, S. E., & Rezanejad-Asl, P. (2024). Psychometric properties of Parenting Sense of Competence Scale using item-response theory. *Heliyon*, *10*(19), e38212. https://doi.org/10.1016/j.heliyon.2024.e38212
